# Epidemiological impact of a syphilis vaccine: a simulation study

**DOI:** 10.1017/S0950268816001643

**Published:** 2016-08-01

**Authors:** D. CHAMPREDON, C. E. CAMERON, M. SMIEJA, J. DUSHOFF

**Affiliations:** 1 School of Computational Science and Engineering, McMaster University, Hamilton, Ontario, Canada; 2 Department of Biochemistry and Microbiology, University of Victoria, Victoria, British Columbia, Canada; 3 Department of Pathology and Molecular Medicine, McMaster University, Hamilton, Ontario, Canada; 4 Department of Theoretical Biology, McMaster University, Hamilton, Ontario, Canada

**Keywords:** Co-infection, HIV, immunization, mathematical modelling, public health, syphilis

## Abstract

Despite the availability of inexpensive antimicrobial treatment, syphilis remains prevalent worldwide, affecting millions of individuals. Furthermore, syphilis infection is suspected of increasing both susceptibility to, and tendency to transmit, HIV. Development of a syphilis vaccine would be a potentially promising step towards control, but the value of dedicating resources to vaccine development should be evaluated in the context of the anticipated benefits. Here, we use a detailed mathematical model to explore the potential impact of rolling out a hypothetical syphilis vaccine on morbidity from both syphilis and HIV and compare it to the impact of expanded ‘screen and treat’ programmes using existing treatments. Our results suggest that an efficacious vaccine has the potential to sharply reduce syphilis prevalence under a wide range of scenarios, while expanded treatment interventions are likely to be substantially less effective. Our modelled interventions in our simulated study populations are expected to have little effect on HIV, and in some scenarios lead to small increases in HIV incidence, suggesting that interventions against syphilis should be accompanied with interventions against other sexually transmitted infections to prevent the possibility that lower morbidity or lower perceived risk from syphilis could lead to increases in other sexually transmitted diseases.

## INTRODUCTION

Syphilis is a sexually transmitted infection (STI) caused by the bacterium *Treponema pallidum*, which affects 36 million individuals worldwide. Every year, it is estimated that 11 million new syphilis infections occur worldwide and 1·5 million pregnancies are affected, putting children at risk of congenital syphilis; in 2008, congenital syphilis caused about 500 000 birth-related adverse outcomes, more than half of them fatal [1, 2]. Most syphilis cases occur in developing countries [3]. After being relatively well controlled in higher-resource countries, syphilis infections have been rebounding since the early 2000s, mostly in high-risk groups [4].

The primary stage of syphilis manifests as a chancre at the infection site. This typically occurs in the genital region and is highly infectious. Weeks to months later, secondary syphilis causes fevers, swollen lymph nodes, and rash; this is the stage when most people present for treatment. Left untreated, or inadequately treated, syphilis can progress to tertiary disease, which can involve the brain, heart, or other organs [5]. Although the incidence of tertiary syphilis has been sharply reduced by treatment, cases of tertiary syphilis are still observed worldwide.

Co-infections involving syphilis and other sexually transmitted pathogens are frequent [6]. Moreover, it has been reported that infection with most STIs (including syphilis) increases the risks of both acquiring and transmitting HIV [7–11]. Given its worldwide prevalence and its shared transmission routes with HIV, syphilis infections may increase HIV incidence [12, 13] either because of an increased susceptibility to HIV or increased HIV infectiousness, although a recent study concluded that the effects of syphilis on incidence via the latter route were small [14]. Although there are limited data for the interaction between syphilis and STIs other than HIV, it is plausible that there also exists a similar epidemiological synergy.

Penicillin has been the main antibiotic used to treat syphilis over the last 70 years. It is inexpensive, readily available in most regions of the world, effective when administered as a single dose during primary, secondary and early latent infection, and *T. pallidum* has thus far not developed resistance against this antibiotic [5].

In contrast, no vaccine is yet available for syphilis [15]. The technical challenges associated with *T. pallidum* experimentation, including its fragility, genetic intractability and unusual ultrastructure, combined with the relative dearth of *T. pallidum* basic science researchers, has impeded the field of *T. pallidum* vaccine research [5, 15]. Sterile protection against challenge with a homologous *T. pallidum* strain has been achieved [16], demonstrating proof-of-concept, but the impractical vaccination regimen used in that study precluded further development as a viable vaccine candidate.

Further factors hindering syphilis vaccine development include the high costs associated with bench-to-bedside vaccine development studies (between $200 and $900 million), the prolonged timeline associated with vaccine development (typically more than 10 years) and, particular to syphilis, the unresolved issues of the target populations, marketability and profitability of a vaccine [15]. However, intensive syphilis-targeted public health control initiatives, including the CDC's National Plan to eliminate syphilis from the United States [17, 18] and the WHO's Initiative for the global elimination of congenital syphilis [19], have not achieved the goal of syphilis elimination, suggesting symptomatic antibiotic treatment alone may not be sufficient to eradicate the disease. Several factors contribute to the difficulty of eliminating syphilis using antibiotic treatment: early infection is difficult to diagnose; varied clinical symptoms lead to mis-diagnoses; diagnostic assays are technically difficult to perform and interpret; an effective and prolonged patient follow-up is required between diagnosis and treatment; and antenatal care may not be available in some settings. These factors lead to missed syphilis diagnoses during the early stage of infection, the highest risk period for transmission and acquisition of additional STIs and congenital syphilis [15]. The continuing high rates of syphilis worldwide, despite the low cost, effectiveness and availability of penicillin treatment, combined with the dire consequences associated with *T. pallidum* infection, especially mother-to-child transmission (MTCT), suggest that alternative means must be used to combat this infection.

The worldwide morbidity caused by syphilis is high, but because eradication through vaccine development will take time and a significant investment of financial and human resources, a careful assessment of the costs and benefits of this option is needed. The WHO's 2013 workshop on ‘Global action plan and roadmap for STI vaccine development and introduction’ highlighted the necessity of mathematical modelling studies to thoroughly compare the costs and benefits of syphilis vaccine development to those of enhanced, targeted screening and treatment programmes [20].

Thus, this study models the epidemiological impact of a potential syphilis vaccine on sexual and vertical transmission of both syphilis and HIV and compares it against an enhanced ‘screen and treat’ programme. We consider heterosexual populations in resource-poor settings using simulations from a detailed computational model.

## METHODS

The epidemiological dynamics of STIs can be challenging: they involve demographics (birth and death rates), sexual behaviour (partnership formation and dissolutions), natural history of disease and potential interactions with other diseases, and population stratification (e.g. risk groups, gender). We developed an agent-based model for this purpose. We hypothesized that syphilis vaccination may have indirect effects on the epidemiology of other STIs, so we chose to model HIV spread along with syphilis because of its high morbidity and availability of co-infection data.

In this section, we highlight the main features of the agent-based model. A full technical description of the model is available in Supplementary file 1.

Our model simulates the partnerships and disease-transmission dynamics of a heterosexual population, along with the natural history of both syphilis and HIV, and their interactions. Individuals enter the modelled population at age 12 years. The general population is stratified, for life, into three sexual risk groups (low, medium and high risk); additionally, females can move in and out of a commercial sex work group.

The contact pattern for STI transmission is driven by partnership formation and dissolution and a rate of sex acts. Individuals can have multiple concurrent partners, and some partnerships are identified as ‘spousal’ (more stable). The decision to form or dissolve any partnership is based on age, age gap with the partner, current number of partners, symptomatic status of potential STIs and risk group. Partnerships and sex-act rates can change at each time step.

The number and type (with or without condom, low or high transmission risk) of sex acts are distributed stochastically, with rates based on age, spousal status, number of concurrent partners, symptomatic status and risk group.

Probabilities of transmission per sex act are specified for both syphilis and HIV, and depend on the duration of infection in the infected partner. Individuals with one STI may be more susceptible to acquiring the other, and co-infected individuals may have increased infectiousness of one or both diseases. Infected individuals can be treated; their adherence to treatment depends on their risk group and probability of treatment success is pre-specified for each STI. In the model, treatment for syphilis instantaneously reduces infectiousness to zero and cures syphilis. Because HIV is not curable and is a chronic disease, treatment is modelled as progressively reducing infectiousness to a value that depends on adherence. With full adherence, HIV infectiousness is progressively eliminated within 3 months of treatment and will remain so as long as adherence is perfect. As adherence deteriorates, infectiousness gradually increases. (See Supplementary file 1 for details.)

**Fig. 1.**
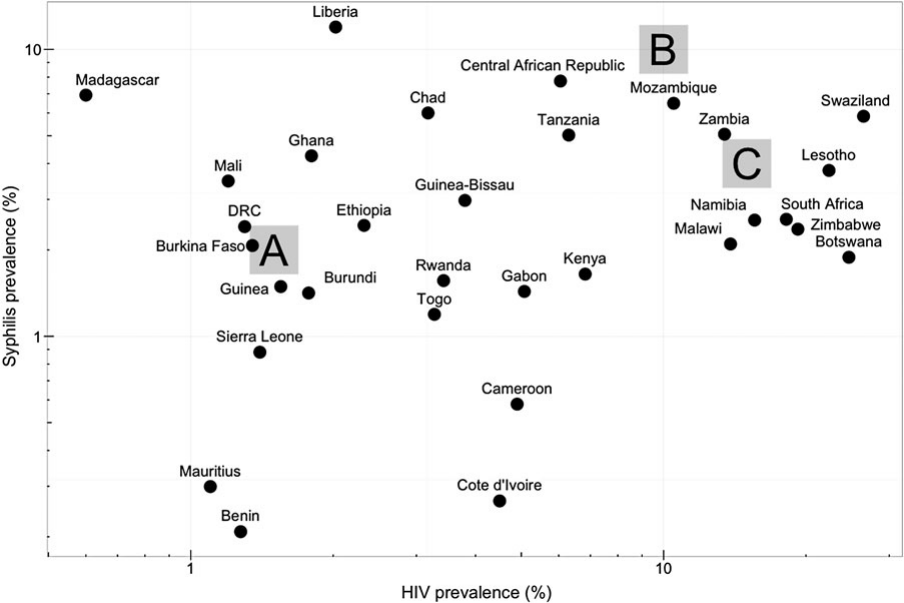
Solid circles (●) are countrywide HIV and syphilis (antenatal care attendees) prevalence estimates for various countries in sub-Saharan Africa [*source*: WHO; prevalence was averaged over available reports ranging between 2001 and 2013 (http://apps.who.int/gho/data/node.main.6177?lang=en)]. Large grey squares represent the prevalence chosen for synthetic populations A–C.

Our study investigates the impact of a syphilis vaccine in settings where syphilis prevalence is higher or equal to the average across sub-Saharan Africa (about 2% [2, 3]). To address heterogeneity among countries, we construct in our agent-based model three synthetic populations (labelled A, B, C) with baseline prevalence of HIV and syphilis representative of scenarios in sub-Saharan Africa (Fig. 1). We do not attempt to represent any specific country. The synthetic populations are exclusively heterosexual and the female-to-male ratio is close to 1.

Demographic characteristics of synthetic populations are based on publicly available data across sub-Saharan Africa. STI prevalences at the population level are fitted by adjusting the baseline treatment rate for each STI and selected behavioural parameters. A complete list of model parameters, their values and their source is provided in Supplementary file 1.

For each synthetic population, we run 30 Monte Carlo iterations to evaluate the epidemiological impact of a hypothetical syphilis vaccination programme compared to symptomatic treatment only. Starting from an initial population of 1250 individuals, the model is run without any STI for 50 years with a coarse time step of 30 days in order to reach equilibrium with the partnership dynamics. By the end of this ‘partnership calibration’ phase, the synthetic population has a size of ~3000 individuals. Then, both syphilis and HIV are introduced and the model is run for 30 more years with a time step of 5 days (the ‘disease calibration’ phase). Prevalence introduction level for syphilis is set as 10%, and for HIV 0·1%, 1% and 2%, for low-, medium- and high-risk groups, respectively; these introductory levels are arbitrarily chosen to be high enough so that there is a small probability of disease extinction early in the simulation; prevalence will eventually converge to their equilibrium values.

**Fig. 2.**
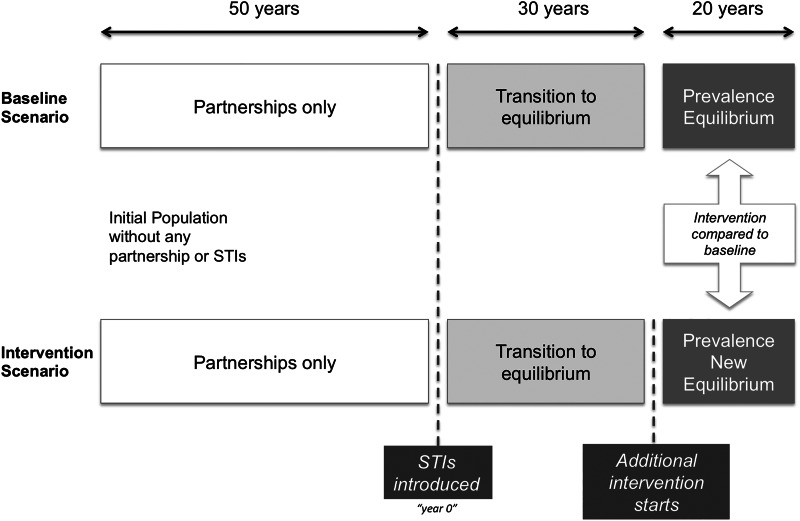
Simulation steps. The model is first run with no sexually transmitted infection (STI) for 50 years in order to reach a steady state in partnership dynamics. Then STIs are introduced and prevalences reach stable values after running the simulation for 30 years. Finally, interventions are introduced and evaluated over a 20-year period.

After the calibration phases, the model is run for an additional 20 years with no changes to provide a ‘baseline’ scenario. We check the calibration steps by confirming that prevalence of syphilis and HIV remain relatively stable throughout this time period. Intervention scenarios are run in exactly the same way as the baseline scenario, except that interventions (increased treatment and/or vaccination programmes) are introduced after 30 years. Hence, intervention scenarios and baseline only differ between years 30 and 50 (Fig. 2).

We assume that susceptibility to HIV is increased about 2·5 times during syphilis infection [8, 13, 21]. To our knowledge, there is little evidence regarding the epidemiological effect of HIV infection on syphilis susceptibility [8]; we assume a 1·5 times increase in our main simulations (sensitivity analysis explored values of 1·0 and 2·5).

We assume that HIV infectiousness increases up to 50% of its maximum possible level (reached during the acute phase) during a syphilis co-infection [7, 22]. Since evidence that HIV can increase syphilis infectiousness is weak, our model does not change syphilis infectiousness when there is a HIV co-infection.

MTCT of syphilis depends on the timing of pregnancy during syphilis infection, being highest when pregnancy occurs early post-infection. We model syphilis MTCT probability with a logistic shape starting at 90% and decreasing to 0% as the duration of syphilis infection increases (Supplementary file 1). The probability of vertical HIV transmission is assumed constant throughout HIV infection and is set at 25%. The three synthetic populations differ by their baseline STI prevalence and treatment, the proportions of the three risk groups, and their partnership rates (Table 1, Supplementary file 1).

**Table 1.** Synthetic population characteristics

Synthetic population	Prevalence HIV	Prevalence syphilis	Syphilis baseline treatment	HIV baseline treatment	Percentage risk groups (low/medium/high)	Partnership maximum rates (formation, dissolution)
A	1·5%	2·0%	Treatment 25% per annum of syphilis + individuals	Treatment (ART) 25% per annum of HIV + individuals	60/35/5	2·0, 0·1
B	10·0%	10·0%	Treatment 1% per annum of syphilis + individuals	Treatment (ART) 15% per annum of HIV + individuals	25/55/20	3·1, 0·4
C	15·0%	4·0%	Treatment 60% per annum of syphilis + individuals	Treatment (ART) 10% per annum of HIV + individuals	35/50/15	3·1, 0·4

ART, Antiretroviral treatment.

We consider four syphilis intervention scenarios (Table 2). The first (labelled ‘TrEnh’) enhances the baseline treatment coverage level: every year, on top of the baseline value, an additional 30% proportion of individuals infected with syphilis, symptomatic or not, are treated. The three other interventions involve a vaccine that is provided to: (i) the whole population at a coverage rate of 10% per annum (‘VaxMass’), (ii) high-risk group individuals and sex workers only at a coverage rate of 20% per annum (‘VaxHiRisk’) and (iii) women aged <18 years only, at a coverage rate of 80% per annum (‘VaxYoung’).

**Table 2.** Description of the simulated interventions

Intervention	Label	Description
Enhanced treatment	TrEnh	Increase baseline mass treatment for syphilis by an additional 30% per annum
Vaccination mass	VaxMass	All sexually active individuals are vaccinated at a rate of 10% per annum
Vaccination high-risk group	VaxHiRisk	Individuals in the highest risk group are vaccinated at a rate of 20% per annum
Vaccination young women	VaxYoung	Women aged ⩽18 years are vaccinated at a rate of 80% per annum

We assume that the failure probability of our hypothetical vaccine is 20% (so that 80% of the vaccinated individuals are perfectly protected immediately after vaccination); values of 0% and 50% were considered in a sensitivity analysis. Vaccine efficacy can wane over time [23], so we assume the modelled syphilis vaccine effectiveness wanes exponentially at a rate of 5% per year (corresponding to 50% loss of effectiveness after 14 years). A sensitivity analysis explored a non-waning vaccine and a rapidly waning vaccine (rate at 70% per year). In the baseline scenario, the vaccine does not provide any infectiousness reduction, but a sensitivity analysis was performed assuming a 50% infectiousness reduction when vaccinated.

We measure the impact of the intervention scenarios described above on both prevalence and vertical transmission of HIV and syphilis, during the 20 years of intervention.

## RESULTS

**Fig. 3.**
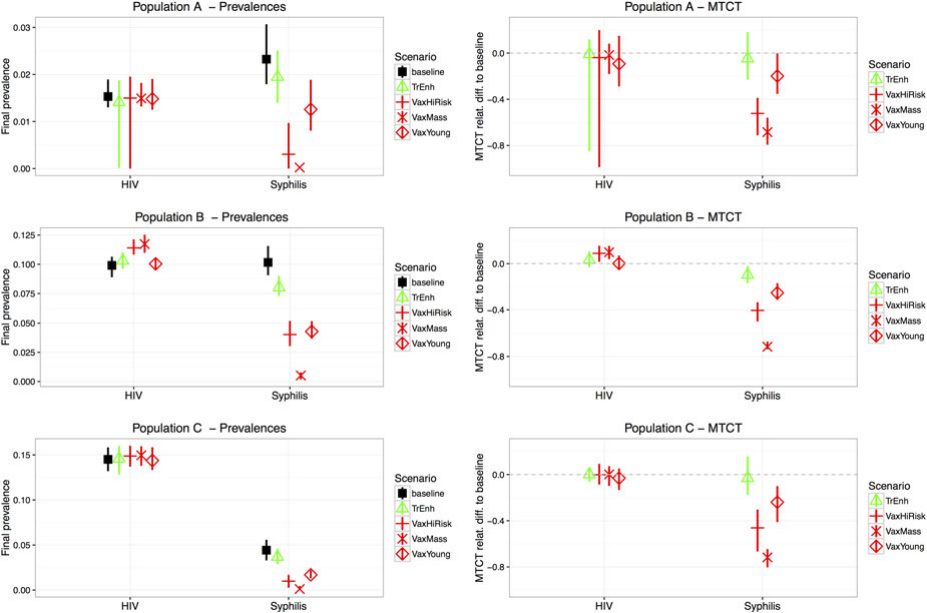
Comparing intervention scenarios. The shape represents the median value and the vertical segment the 10–90% quartile range calculated from 30 Monte Carlo simulations. Panels on the left-hand side show final prevalence of HIV and syphilis for all modelled interventions in three synthetic populations. Prevalence is calculated after 20 years of intervention. Right-hand side panels show the relative difference of cumulative mother-to-child syphilis transmission (MTCT) compared to the baseline scenario (horizontal dashed line at 0).

Within each of the three synthetic populations (A, B, C), the five scenarios (baseline and four additional interventions) were run and compared. The results from the Monte Carlo simulations for final STI prevalences and MTCT rates are summarized in Figure 3.

Overall, for all synthetic populations, the most successful intervention to reduce syphilis prevalence is mass vaccination (VaxMass): all simulations show extremely low levels of prevalence after 20 years. Indeed, in this intervention scenario, the median syphilis prevalence is reduced to <0·01% in all three synthetic populations. Vaccinating the high-risk population (VaxHiRisk) and targeting young females only (VaxYoung) also leads to significant reduction in overall syphilis prevalence. Increasing coverage for syphilis treatment (TrEnh) reduces overall syphilis prevalence much less (Fig. 3, Supplementary file 2).

MTCT of syphilis is also reduced significantly over the 20-year intervention period especially with the mass and high-risk vaccination strategies (Fig. 3, Supplementary file 2).

HIV prevalence is not affected in populations A and C, and slightly increases in population B during the vaccination interventions targeting the general population (VaxMass) and high-risk groups (VaxHiRisk). The changes in HIV vertical transmission mirror those observed for prevalence (Fig. 3, Supplementary file 2).

The results from the sensitivity analysis (described in the Methods section) do not substantially change the qualitative conclusion drawn from the central scenarios (see Supplementary file 3).

## DISCUSSION

The effect of a hypothetical syphilis vaccine on the burden of syphilis disease, in particular its congenital form, is expected to be large. The potential effect on HIV is less straightforward.

We used an agent-based model to simulate the horizontal and vertical spread of both syphilis and HIV in various syphilis vaccination scenarios 20 years after their introduction. We modelled synthetic populations representative of settings found in sub-Saharan Africa.

As expected, we found, regardless of the synthetic population considered, that a hypothetical syphilis vaccine could eliminate or markedly reduce incidence of congenital and sexually acquired syphilis when vaccination strategies target the whole population (mass vaccination) or focused on high-risk groups. Moreover, targeting young females appeared much less effective over the studied time horizon of 20 years, because the older cohorts that are not eligible for vaccination continue to spread infections over many years.

Given that both syphilis and HIV are suspected to increase the transmission of the other through various mechanisms, we hypothesized that a syphilis vaccine might indirectly reduce HIV incidence. However, in our modelling framework, we found that HIV was largely unaffected by syphilis vaccination, except that the high syphilis-prevalence population (B) experienced a small *increase* in HIV transmission in response to syphilis vaccination. This is because symptomatic syphilis infections in our model are associated with reduced sexual activity: thus, reducing syphilis will lead to an increase in sexual activity, particularly in high-risk groups. This effect is noticeable when a large proportion of individuals are involved in risky sexual behaviour and syphilis prevalence is high. Hence, if a syphilis vaccine is developed, vaccination programmes should be accompanied with intensified interventions on transmission of other STIs.

This predicted effect from our simulations is similar to an observed phenomenon, with syphilis and HIV exchanging roles. Syphilis and other STIs have increased in many populations since an effective HIV antiretroviral therapy became available in the early 2000s (documented especially in men who have sex with men in western countries, see [24] for example). A possible cause for this increase is less fear of acquiring HIV, and less fear of HIV disease, leading to increased sexual risk behaviours.

Our simulation results may also shed light on several large clinical trials that targeted curable STIs in an attempt to reduce HIV transmission [25–32]. Most of these trials did not show a significant reduction in HIV incidence in the treated arm. General and trial-specific interpretations to these unexpected outcomes have been proposed [33]. Here, our study suggests another possible mechanism: if intensity of sexual activity is affected by (symptomatic) STI episodes, curing some STIs could increase sexual activity, and thus increase incidence of non-treated STIs.

Our simulations also suggest that high HIV prevalence does not hamper syphilis vaccination programmes at the population level. This result is robust to vaccine failure at 50%, assuming that failure is not individual-dependent (see sensitivity analysis in Supplementary file 3). We did not, however, consider the possibility that HIV infection increases syphilis vaccine failure.

Our study has several limitations. Our results are based on synthetic populations, so their practical translation to real communities may not be straightforward. However, we chose the demographic and behavioural characteristics of the three synthetic populations to be similar to what can be found across sub-Saharan Africa, so we expect some relevance when applied to real communities in resource-limited countries.

Our model does not account for birth control or abortion. If high-risk groups are more likely to employ these, we may be overestimating vertical transmission at the population level. We therefore chose to present vertical transmission in relative rather than absolute terms in Figure 3.

We restricted our study to the epidemiological impact of introducing a syphilis vaccine. A natural extension of our work would be a cost-effectiveness analysis.

In summary, our results suggest that a syphilis vaccine has the potential, over a 20-year horizon, to eradicate horizontal and vertical transmission of this disease in populations with various levels of baseline syphilis and HIV prevalence and risk behaviours, while expanded treatment interventions are likely to be substantially less effective. Vaccination programmes targeting the whole population and/or high-risk groups achieve significantly better incidence reduction than when targeted at young women only. Our results also highlight that syphilis vaccination programmes should be accompanied with intensified interventions on other STIs in order to prevent possible incidence rebounds caused by decreased symptoms or lower perceived risk, following syphilis vaccination.

## Supplementary material

For supplementary material accompanying this paper visit https://doi.org/10.1017/S0950268816001643. click here to view supplementary material
