# Supplementary material for: Epidemiological impact of a syphilis vaccine: a simulation study
Source: Epidemiol Infect. 2016 Aug 1;144(15):3244–52. doi: 10.1017/S0950268816001643 (PMC5080673; doi:10.1017/S0950268816001643)
Supplement: Supplementary file 1 [file hygsup.zip › S0950268816001643sup001.pdf]

STI-Agent documentation

David Champredon

June 30, 2016

Forewords

This document describes the mathematical model used in the following article:

Epidemiological impact of a syphilis vaccine: a simulation study.
D. Champredon, C. Cameron, M. Smieja and J. Dushoff.

The computer code is available at https://github.com/davidchampredon/syphilisvax. Please note that a significant computing power (more than 12 CPUs at 3 GHz) is suggested to run simulations on populations larger than 5,000 individuals.

Contents

1 Introduction and Summary 4

2 Individuals, Population and STI objects 5

3 Demographics 5
- 3.1 Birth . . . 5
- 3.2 Death . . . 6

4 Partnerships 6
- 4.1 Partnerships formation . . . 6
  - 4.1.1 Formation algorithm . . . 7
  - 4.1.2 Formation success random variable (Φ) . . . 8
- 4.2 Spousal union . . . 9
  - 4.2.1 Determinants . . . 9
  - 4.2.2 Algorithm . . . 10
- 4.3 Partnerships dissolution . . . 11
  - 4.3.1 Dissolution algorithm . . . 11
  - 4.3.2 Dissolution success random variable (Ψ) . . . 11
- 4.4 Number of concurrent partners . . . 12

5 Sexual intercourses 13
- 5.1 Total number of sex acts . . . 13
- 5.2 Distribution of sex acts among partner types . . . 14
- 5.3 Distributing the number of sex acts between partners . . . 15
- 5.4 Distributing sex acts types . . . 15
- 5.5 Limits on the number of sex acts for females . . . 16
- 5.6 Sex acts of males with no partnership . . . 16

6 Commercial sex workers 17
- 6.1 Recruitment . . . 17
- 6.2 Cessation . . . 17

7 Disease transmission 18
- 7.1 Infectivity curve and susceptibility factor . . . 18
- 7.2 Probability of transmission . . . 18
- 7.3 Probabilities of transmission for every STI . . . 19
  - 7.3.1 HIV . . . 19
  - 7.3.2 Syphilis (Treponema pallidum, Tp) . . . 20
- 7.4 Mother-to-child (vertical) transmission . . . 22

8 Treatment and vaccination 23
- 8.1 Treatment implementation . . . 23
  - 8.1.1 Treatment microbiological failure . . . 23
  - 8.1.2 Adherence . . . 24
  - 8.1.3 Treatment reduction effect . . . 24
- 8.2 Vaccine implementation . . . 24

9 Calibration 25

10 Simulations 26

11 Appendix 27

11.1 Pseudo-beta shape function 27
11.2 Tables of all parameters 27

1 Introduction and Summary

This documentation describes the implementation of a stochastic individual based epidemiological model that studies specifically sexually transmitted infections (STIs). A particular aim of this model is its ability to simulate epidemics of several concomitant STIs. This section gives an overview of the main features of the model, without giving any technical details.

This model attempts to represent fairly realistically three dynamics:

- **Demography:** Some sexually transmitted infections (*e.g.*, HIV, HSV2, Syphilis) have an infectious period that lasts years if not decades. So, unlike other diseases (*e.g.*, influenza) where the demographic changes over the epidemic period could be neglected, here it is important to have a good representation of the ageing processes of the population (growth rates but also age distribution). Individuals are simulated from the age of sexual debut (for example 12 years old) to an age where sexual activity is very unlikely ('maximum age', for example 80 years old). Pregnancies stochastically starts following intercourses and determine births. Death can occur naturally or can be disease induced (both distributed as Weibull). When an individual reaches the maximum age, death is provoked. There is no migration in or out the population (apart from recruitment of commercial sex workers).
- **Sexual activity:** The contact pattern for STI transmission is driven by partnership formation/dissolution and the rate of sex acts. Individuals can have multiple concurrent partners. Spousal partnerships are also modelled: they are a non-negligible fraction of all partnerships and their formation/dissolution process is distinctive from casual ones. The decision to form or dissolve any partnership is based on stochastic events following rules based on age, current number of partners, symptomatic status of potential STI infections and risk group. There are three risk groups for the general population: low, medium and high-risk. Individuals belonging to a given risk group will be assigned representative parameter values associated with their risk behavior (*e.g.*, use of condom, number of concurrent partners, partner switch rates, etc.). High activity commercial sex workers (engaging with multiple partners in a very short period of time) form a fourth distinct risk group with a specific partnership formation process. The rate and type of sex acts are distributed randomly based on several variables (*e.g.*, age, spousal status, number of concurrent partners, symptomatic status, etc.).
- **Disease transmission:** Once the network of partnership is built following the demographic and partnerships processes, diseases can spread through the population. STIs have very different infectious features: probability of transmission per sex act, infectious period and recurrence frequency can vary of orders of magnitudes. Hence, each STI is represented with its own infectivity curve (probability of transmission with respect to time). There are adjusting coefficients on the level of the infectivity curve simulating a potential increase in infectiousness from an individual infected with another STIs. Likewise, susceptibility to STIs varies with potential co-infections.

The simulations are run in three steps. First, a simulation starts from an initial population with no partnerships and the model runs for a long enough time (typically 50 years with a coarse time step of a month) to match target levels on demography (for example growth rate and age distribution) and partnerships (fraction of single individuals, fraction of spousal partnerships). This is the pre-epidemic era where the model should reach its equilibrium values. The second step introduces STIs in the population. The model is run for a long enough with a fine time step (typically of the order of a day), in order to have a good fit with target prevalences of the epidemic era. The third step is the analysis and/or prediction. Simulations are run with intervention strategies and/or introduction of a new STI.

The model is implemented in C++ and wrapped in a R library[1]. Computing power is critical when the population is large and the time horizon long (typically more than 10,000 individuals for more than 10 years with a time step shorter than a week), so a basic parallel implementation is used.

For the reader interested in looking into the C++ and R computer codes, it is available here: https://github.com/davidchampredon/syphilisvax.

2 Individuals, Population and STI objects

It is important to note there are three main classes of C++ objects: `Individual`, `Population` and `STI`. In this individual-based model, a distinction is made between attributes at the 'atomic' individual (i.e., age) and the ones that belong to the population (i.e., maximum partnership formation rate).

An individual is mainly characterized by biological and social features (this is *not* an exhaustive list):

- biological: gender, age, STI infections, ...
- social: risk group, number of partnerships, marital status, ...

A population is characterized by a vector of individuals, a vector of STIs and other scalar parameters (like, for example, the maximum rate of partnership formation).

STIs objects describe the key features of the natural history of the infection. It is independent of individuals or populations.

3 Demographics

3.1 Birth

After every sexual contact, the chance a female gets pregnant is determined by a Bernoulli random variable and its probability (becoming pregnant per sex act) is a model parameter. All new borns and their potential acquired infection are recorded (so that we can keep track of MTCT incidence in a simulation). Children between birth and minimum age of sexual activity are not modelled. Young individuals just turning the minimum age of sexual activity enter randomly the population. The expected rate of arrival is

$$\alpha = \text{birthRate} \times (1 - m_{\text{infant}})(1 - m_{\text{child}})^4(1 - m_{\text{child}}/2)^{n_{min}-5}$$

with m_{infant} the mortality rate of infant ($<$ 1 year-old), m_{child} the mortality rate of children $<$ 5 years-old and n_{min} the minimum age of sexual activity (it is assumed the mortality rate for children between 5 and n_{min} is half).

Then, the number N of youth (aged n_{min} years-old) arrivals during a given period of time dt, for a population of size T, is:

$$N \sim \text{Poisson}(\alpha \, T \, dt)$$

3.2 Death

The classical methodology of survival analysis is applied here. The probability of dying between this time step and the previous one is assessed for every individual, at every time step. The probability driving this process is based on

$$\Pr(t < T_{\text{death}} < t + dt \mid T_{\text{death}} \geq t) = \frac{F(t+dt) - F(t)}{1 - F(t)}$$

where T_{death} is the time of death and F its cumulative distribution function. It is assumed that the survival time is Weibull distributed, so the associated hazard function h is given by

$$\begin{aligned} h(t) &= \lim_{dt \to 0} \Pr(t < T_{\text{death}} < t + dt \mid T_{\text{death}} \geq t)/dt \quad (1) \\ &= k\lambda(\lambda t)^{k-1} \quad (2) \end{aligned}$$

for $k > 0$ and $\lambda > 0$ the standard Weibull shape and scale parameters, and t the age of the individual.

If t_{HIV} is the time when the individual was infected with HIV, the hazard function changes and is now parameterized with new shape and scale parameters, and duration since infection instead of age:

$$h_{HIV}(t) = k'\lambda'(\lambda'(t - t_{HIV}))^{k'-1}$$

This probability has to be evaluated at every time step, for every individuals (for example, if HIV treatment is initiated, the associated hazard will decrease). An example of a plot of this hazard function is illustrated in Figure 1.

4 Partnerships

4.1 Partnerships formation

Let's define r^* the maximum annual rate of consideration to form partnership and F (resp. M) the population size of females (resp. males) with a partnership deficit. A partnership deficit is defined, for a given individual, as the maximum possible partnerships minus the number of concurrent partnerships. The unit of r^* is time^{-1}.

Females and males who do not have a partnership deficit are - by definition - not available to form new partnerships, hence are ignored right from the start of the formation process.

It is assumed that the maximum number of partnership formations during a unit time period is given by [2]

$$P^* = r^* \frac{FM}{F + M}$$

For sake of clarity, if we assume that every consideration will lead to a partnership formation and note P the total number of partnerships, we have:

$$\begin{aligned} \frac{dF}{dt} &= -r^* \frac{FM}{F+M} = -\left(r^* \frac{M}{F+M}\right) F = -r_f^* F \\ \frac{dM}{dt} &= -r^* \frac{FM}{F+M} = -\left(r^* \frac{F}{F+M}\right) M = -r_m^* M \\ \frac{dP}{dt} &= \frac{1}{2} 2r^* \frac{FM}{F+M} = r^* \frac{FM}{F+M} \end{aligned}$$

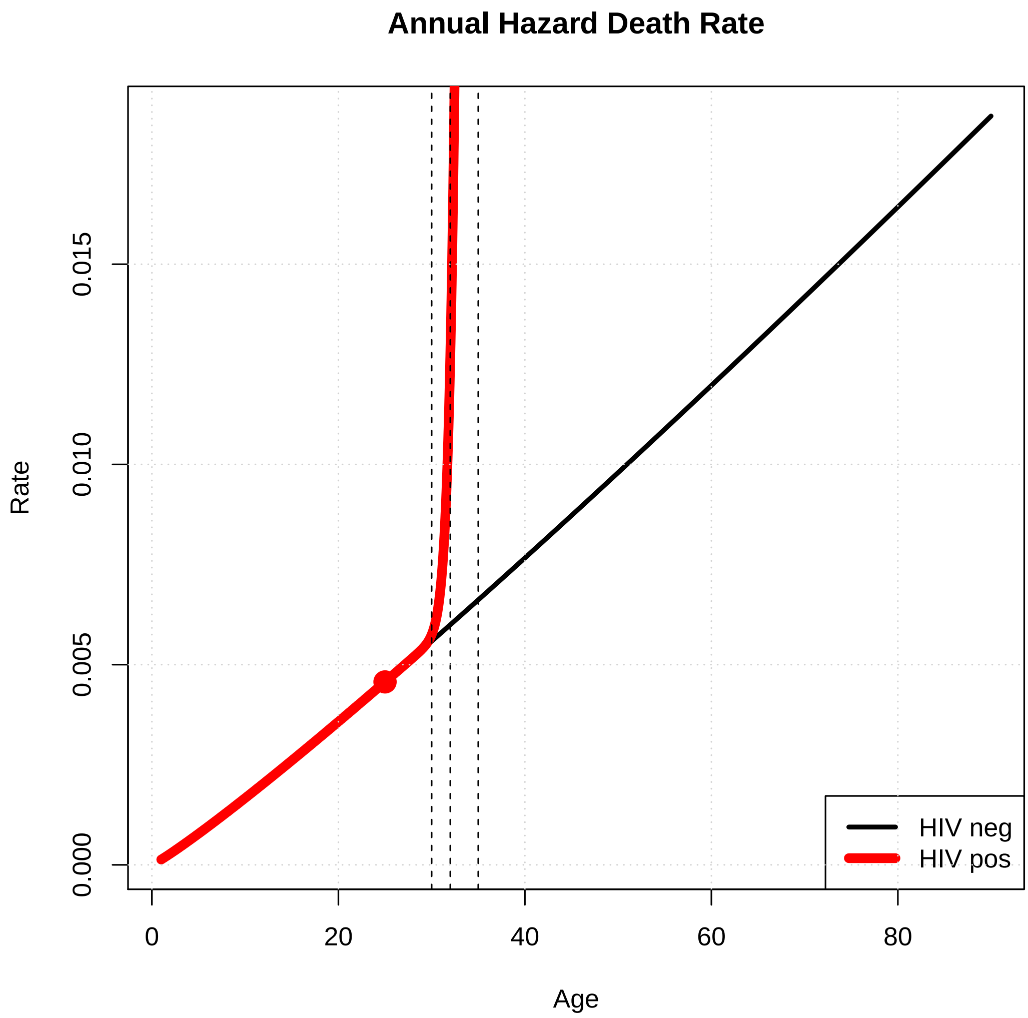

Figure 1: Death hazard. The red dot represents the age of HIV acquisition. Vertical dashed lines are set at 5, 7 and 10 years after HIV acquisition.

Hence, r_f^* is interpreted as the rate of partnership formation when female dominance is assumed. The formulation is symmetrical if male dominance is assumed, so we'll assume female dominance.

4.1.1 Formation algorithm

The partnership formation process is stochastic and driven by the algorithm below.

1. Calculate $F^* \sim \text{Binomial}(r_f^* \, dt, F)$, the maximum number of females candidate for partnership formation during the period dt
2. Pick randomly F^* females among the F available females. Collect and store their positions in the population in the set $\mathcal{S}_f$.
3. For each female in $\mathcal{S}_f$, pick randomly an available male
4. Draw the binary random variable Φ that determines if this pair will form a partnership ($\Phi = 1$ means formation success). See 4.1.2 for the distribution of Φ.

5. If formation success on this pair ($\Phi = 1$), then form partnership[1]. Else, do nothing.
6. This female is removed from the pool of partnership candidates (whether formation was successful or not): update $\mathcal{S}_f$ accordingly by deleting her position.
7. If $|\mathcal{S}_f| > 0$, go to step 3; else stop.

4.1.2 Formation success random variable (Φ)

Given two candidate individuals, I_m (male) and I_f (female), the success of formation is determined by the binary random variable $\Phi \sim \text{Bernoulli}(p)$. When $\Phi = 1$, the two candidates do form a partnership.

The probability of success to form the partnership depends on several tests on variables from both individuals (age, risk group, etc). Hence, it is assumed the probability of a successful partnership formation between the two candidates is given by

$$\Pr(\Phi = 1) = f_{age}\ f_{risk}\ f_{\text{deficit}}\ f_{STI} \tag{3}$$

where all functions f are valued in the interval $[0;1]$ and are defined hereafter.

- **Age.** The age A of an individual determines its attractiveness to the opposite sex. The age gap between the candidate male and female is defined as $G = A_m - A_f$. Then, the age component of the rate of couple formation is assumed to depend on the age and marital status of the female, and the age gap with the candidate male:

$$f_{\text{age}} = \varphi(A_f, G)$$

The function φ describes the joint distribution of the female age (a) and age gap (g):

$$\varphi(a, g) = e^{-s_{\text{age}} X^T M^{-1} X}$$

with $X = (a - \bar{a}, g - \bar{g})^T$ the (centred) vector for female age and age gap, $\bar{a}$ (resp. $\bar{g}$) the average age (resp. age gap) at partnership formation, s_{age} a shape parameter, and M the covariance matrix

$$M = \begin{pmatrix} \sigma_a^2 & \rho\sigma_a\sigma_g \\ \rho\sigma_a\sigma_g & \sigma_g^2 \end{pmatrix}$$

Parameters σ represents the variance of the ad-hoc variable and ρ the correlation between female age and age gap, and can be calibrated on DHS data.

- **Risk group.** Both candidate individuals belong to a risk group, r_m and $r_f \in \{0, 1, ..., r^*\}$, where r^* is the highest risk group. The candidate couple's risk score is $r_f + r_m$. The probability component regarding the risk group is

$$f_{risk}(r_f, r_m) = e^{-s_0^{\text{risk}}(2r^* - (r_f + r_m)) - s_1^{\text{risk}}(r_f - r_m)^2}$$

where s_0^{risk} and s_1^{risk} are shape parameters that should be fitted globally (no specific data). Note that when both partner belong to highest risk group ($r_f = r_m = r^*$), then $f_{risk} = 1$ and when both belong to the lowest $f_{risk} = e^{-2r^* s_0^{\text{risk}}}$.

[1] `Population.formPartnership(i,j)` is called

- **Partnerships deficit.** Define n_f as the number of concurrent partnerships for the candidate female considered, n_f^* her maximum number of partnerships, $d_f = n_f^* - n_f$ the deficit number of partnerships and deficit ratio $D_f = d_f / n_f^*$. Same notations for males. The probability component regarding the partnership deficit is

$$f_{deficit}(n_f, n_f^*, n_m, n_m^*) = (D_f D_m)^q$$

  with $q \geq 1$ a parameter to calibrate globally.

- **STI infection.** If an individual has a symptomatic STI infection, the likelihood to form a partnership is reduced. Symptoms can be painful, reducing the willingness to engage in a sexual contact. Symptoms can be visible (especially in males), reducing the attractiveness of a sexual contact. Define $s_f \in \{0, 1\}$ the variable signalling a symptomatic infection with *any* STI within the candidate female partner, and $a_{sympt,f}$ the relative reduction of the probability that a partnership can be formed in the presence of these symptoms. Same notations for males. Values for parameters a_{sympt} will have to be assumed (not calibrated).

  Note: Repulsion of STI symptoms may not be the same for all STIs in reality. This feature can be considered for a future development.

  The probability component regarding the STI infection is

$$f_{STI}(s_f, s_m) = \left(a_{sympt,f} \mathbf{1}_{s_f=1} + \mathbf{1}_{s_f=0}\right) \left(a_{sympt,m} \mathbf{1}_{s_m=1} + \mathbf{1}_{s_m=0}\right)$$

  where the product is over all STIs modelled.

4.2 Spousal union

A spousal union is defined as a partnership that has been celebrated under the civil or religious law. The reason to model this special partnership is to reflect the facts that such a relationship is likely to have a higher sexual intercourses frequency and is more difficult to dissolve because of social pressures.

4.2.1 Determinants

It is assumed that all partnerships starts as casual relationships that can evolve as a spousal union. At every time steps, based on several parameters (described hereafter) all the partnerships a male has are re-assessed to become a spousal union[2].

The spousal progression rate is assumed to be driven by the age of the female (A_f), her age gap G with the potential husband, her current marital status m, the number n of existing wives the male already has, the difference between the age gap G and the ones of existing wives (if any), and finally the duration of this casual partnership.

The rate of spousal union formation, noted S, is assumed to have the following functional form:

$$S(A_f, G, \tau, m) = B. \left[\mathbf{1}_{n=0} + K\, \mathbf{1}_{1 \leq n < n_{\max}}\right] . d(\tau).sp^* \qquad (4)$$

With sp^* the maximum rate of spousal progression. Functions B, K and τ are define hereafter.

[2] This can be a limitation in the context of arranged marriage where the partnership starts as a spousal one right from the start (no prenuptial period). To mitigate this limitation, the rate of spousal union can be very high such that spousal determination occurs almost instantaneously.

Age and age gap
Function B represents the probability the spousal transition is successful based on the female's age, age gap and her current marital status m:

$$B = s(A_f, G)$$

$$s(A_f, G) = \mathcal{N}(A_f, \bar{A}_f, \sigma_{A_f})\mathcal{N}(G, \bar{G}, \sigma_G)$$

where $\bar{A}_f$ (resp. $\bar{G}$) is the average age (resp. age gap) of a female entering her first union, and $\mathcal{N}(x, m, \sigma) = \exp(-(x-m)^2/2\sigma^2)$ If the female is already in a spousal union, then she cannot be considered to be a spouse of another man (polygynous population).

Note: Future development will consider other shapes for $\mathcal{N}$, as DHS data do not fully support the one chosen.

Gaps with other spouses
Function K reflects the fact that if a female enters an existing polygynous union, the age gap with the new comer (G) is more likely to be larger than with existing wives ($G_1, ..., G_n$):

$$K = K(G_1, ..., G_n, G) = e^{-(\Delta-\bar{\Delta})^2/2\sigma_\Delta^2}$$

$$\Delta = \min(G_1, ..., G_n) - G$$

Duration of partnership
It is assumed the rate of progression to a spousal union changes with the duration of this partnership τ, and is represented by the function d:

$$d(\tau) = \mathcal{N}(\tau, k_1, k_2)$$

with k_1 (average partnership duration when spousal progression occurs) and k_2 (variance) constants to be fitted globally.

Summary of all spousal progression parameters:

- n the number of existing spouse(s) this male currently has, and $n_{\max}$ the maximum number of spouses this male can ever have
- τ the duration of this partnership
- $d(\tau)$ represents the probability of spousal conversion with respect to duration of this partnership
- m the current marital status ("never coupled (nc)", "uncoupled separated (us)") of the *female*
- $\Delta = \min(G_1, ..., G_n) - G$ the difference of age gaps between the youngest existing wife and the candidate wife; its mean is noted $\bar{\Delta}$ and its variance σ_Δ. Both can be calibrated on DHS data

4.2.2 Algorithm

At each time steps:

1. Select one male with at least one casual partnership
2. Loop on all casual partnerships

3. Calculate S_i, the rate of spousal progression of the ith casual partnership, using Equation (4)
4. Draw the Bernoulli random variable $\mathcal{S}$ with rate S_i. If $\mathcal{S} = 1$, then upgrade this casual partnership to a spousal union; else do nothing
5. Go to step 1 until all males with at least one casual partnership have been scanned

4.3 Partnerships dissolution

Dissolutions of partnerships follows the same idea as their formation. A maximum annual rate of dissolution per partnership, δ^* (unit is time^{-1}), is assumed for the whole population. The total number of partnerships is noted P. This gives a maximum number of candidate partnerships for dissolution. If all dissolution considered would actually dissolve the partnerships, the evolution of the number of partnerships would be given by $P' = -\delta^* P$. However, the success of dissolution will be determined by the characteristics of both individuals forming this partnership.

4.3.1 Dissolution algorithm

The dissolution process is described by the following stochastic algorithm.

1. Calculate $P^* \sim \text{Binom}(\delta^* dt, P)$ the maximum number of partnerships considered for dissolution during the period dt
2. For each partnership, draw the binary random variable Ψ that determines if this partnership will be successfully terminated. See 4.3.2 for the distribution of Ψ.
3. If dissolution is successful ($\Psi = 1$), then dissolve this partnership. Else do nothing.
4. If at least one partnership candidate for dissolution remains, go to step 2; else stop.

4.3.2 Dissolution success random variable (Ψ)

Given a candidate partnership composed of two individuals, I_m (male) and I_f (female), the success of dissolution is determined by the binary random variable $\Psi \in \{0, 1\}$. When $\Psi = 1$, this candidate partnership is dissolved. The Bernoulli probability for Ψ is function of several variables, described below.

- **Spouse.** Dissolving a spousal partnership is less likely because of social pressures. Define the binary variable s indicating if this partnership is a spousal one. The probability component regarding spousal relationship is

$$g_{spouse}(s) = \epsilon \mathbf{1}_{s=1} + \mathbf{1}_{s=0}$$

  with $0 < \epsilon < 1$ a parameter to calibrate globally.

- **Relationship duration.** Define d the duration of the candidate partnership. It is assumed that short partnerships are more likely to dissolve than the ones that have survived for a longer time. The probability component regarding relationship duration is

$$g_{duration}(d) = \text{dur}_1 + \text{dur}_2\, e^{-\text{dur}_3\, d}$$

  with $0 < \text{dur}_1, \text{dur}_2 < 1$ and $\text{dur}_3 > 0$ parameters to be fitted globally.
  Note $\text{dur}_1 + \text{dur}_2$ is the probability of dissolution just after a time unit (e.g. one day), hence this models a "one-off" contact.

- **Partnerships deficit** The probability this partnership dissolves is assumed to be decreasing as the partnership deficit of both members increases. Define n_f as the number of concurrent partnerships for the female, n_f^* her maximum number of partnerships, $d_f = n_f^* - n_f$ the deficit number of partnerships and deficit ratio $D_f = d_f / n_f^*$. Same notations for males. The probability component regarding the partnership deficit is

$$g_{deficit}(n_f, n_f^*, n_m, n_m^*) = q_{min} + (1 - q_{min})((1 - D_f)(1 - D_m))^q$$

  with $q \geq 1$ a shape parameter and q_{min} the minimum contribution of this component.

- **Risk group.** Both candidate individuals belong to a risk group, r_m and $r_f \in \{0, 1, ..., r^*\}$, where r^* is the highest risk group. The candidate couple's risk score is $r_f + r_m$. The probability component regarding the risk group is

$$g_{risk}(r_f, r_m) = e^{(r_m + r_f - 2r^*)\mathrm{drsk}_1}$$

  with drsk_1. This parameter should be fitted globally (no specific data).

- **Age.** Define A_f and A_m the age of the female and male in the partnership candidate for dissolution. The probability to dissolve the couple is assumed to decrease with the "couple age" $A_f + A_m$ and also depends on the age gap. The probability component regarding ages in this relationship is

$$g_{age}(A_f, A_m) = e^{-(A_f + A_m - \mathrm{dage}_1)^2 / \mathrm{dage}_2}$$

  with dage_1 is the average couple age where dissolution risk is maximum and dage_2 its variation. These parameters are fitted globally.

- **STI symptoms.** If one of the member of the candidate partnership has a symptomatic STI, this can increase the risk of terminating this partnership. Define $s \in \{0, 1\}$ the variable signalling a symptomatic infection in a given partnership and $0 < d_{sympt} < 1$ the relative reduction of the probability to dissolve in the abscence of these symptoms. The probability component regarding the STI infection is

$$g_{STI}(s) = \mathbf{1}_{s=1} + d_{sympt}\mathbf{1}_{s=0}$$

  Note: Some STIs may exhibit more 'repulsive' symptoms, but for now treat all STIs the same way.

Similarly as with the formation process, putting everything together, the probability of a successful partnership dissolution is

$$\Pr(\Psi = 1) = g_{spouse}\ g_{age}\ g_{risk}\ g_{duration}\ g_{deficit}\ g_{STI} \tag{5}$$

4.4 Number of concurrent partners

The maximum number of concurrent partners, n, is determined for each individual, based on its risk group. It is assumed to have a geometric distribution:

$$n \sim \mathrm{Geom}(p)$$

$$p = c_1 e^{-c_2 r}$$

with c_1 and c_2 gender-dependant shape parameters, and r the risk group of the individual.

5 Sexual intercourses

The rate of sexual intercourses in a partnership is assumed to be driven by the male. Although females can have some negotiating power regarding partnership formation and continuation, they seem less able to control sexual practices once in a partnership [3].

Following how partnerships are modelled, there are three categories of sex partners:

- Spouses
- Casual partners
- Sex workers

Three types of sex acts are modelled here:

- sex act with condom
- sex act without condom, "low risk" practices (i.e. vaginal) that do not increase the risk of HIV or STI transmission
- sex act without condom, "high risk" practices (i.e. anal, dry-sex) that increase the risk of HIV or STI transmission

A male will have a specified number of sex acts during a period of time. The model will distribute these sex acts between all different partners and assign them the type of sex act, based on binomial distributions. This is described hereafter.

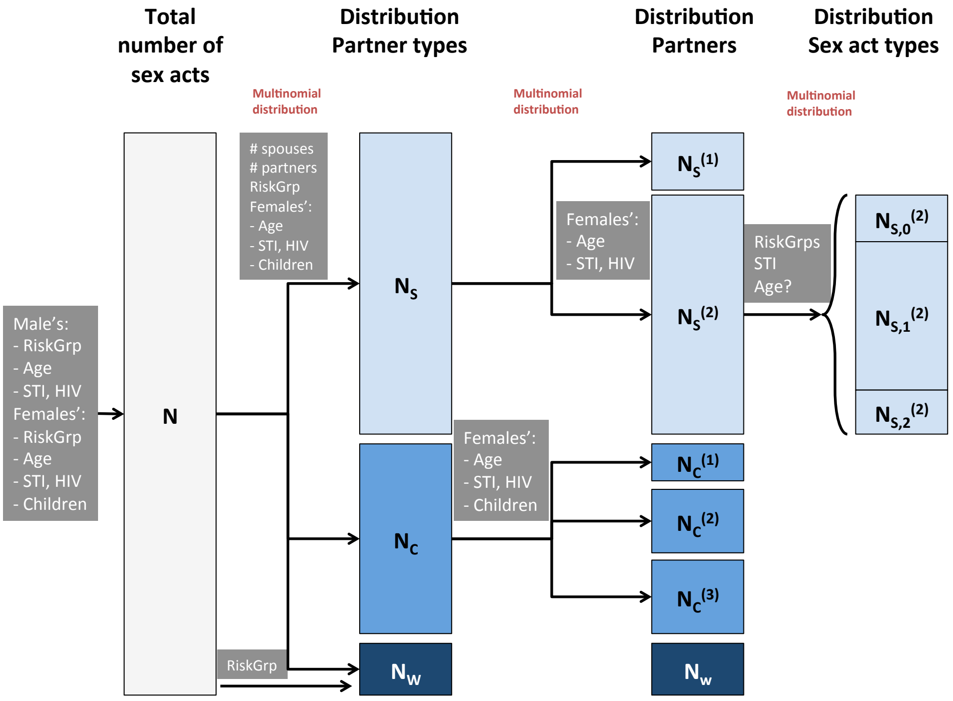

Figure 2: Distribution of number and type of sex acts among partners

5.1 Total number of sex acts

A male has a rate of sexual intercourses with *any* partners (spousal, casual or sex worker) noted R_{sex} and the actual total number of intercourses performed by this male, N, during a period dt

is distributed with a Poisson distribution:

$$N \sim \text{Poisson}(R_{\text{sex}}\, dt)$$

The rate R_{sex} is set at a starting value, R_{sex}^{max}, the maximum rate of sexual intercourses for any male (think of it as a biological limit). Then, this rate is reduced by a factor R_M depending on the male's features and another factor R_F depending on his partners' features:

$$R_{\text{sex}} = R_{\text{sex}}^{max} \times R_M \times R_F$$

Factor R_M depends on the male's age A_m, his risk group r, if he has symptoms of any STI (binary variable $s = 0$ if no symptoms), the total number of partners n. The functional form is defined as:

$$R_M = h_{age}(A_m)\, h_{risk}(r)\, h_{STI}(s)\, h_{nPartn}(n)$$

Similarly, R_F is defined as

$$R_F = h_{age}(\bar{A_f})\, h_{risk}(\bar{r})\, h_{STI}(\bar{s})$$

where $\bar{A_f}$ is the average age of all male's partners, and similar notation for other variables.

Then, we define each function h corresponding to the associated determinant.

$$h_{age}(a) = \exp\left(-\left(\frac{a - a_{peak}}{\sigma_{age}}\right)^q\right)$$

with a_{peak} the mean age of peak sexual activity (at the population level) and σ_{age} and q shape parameters. These parameter may have to be assumed if no relevant data set found.

$$h_{risk}(r) = (1 - \epsilon_{risk})\frac{r}{r^*} + \epsilon_{risk}$$

with r^* the maximum risk group and ϵ_{risk} representing the fraction of sex acts a male in the lowest risk group has compared to the highest one.

$$h_{STI}(s) = \begin{cases} \epsilon_{STI} & \text{if } s = 1 \\ 1 & \text{if } s = 0 \end{cases}$$

with ϵ_{STI} representing the fraction of sex acts performed when individuals have STI symptoms. To reflect the unbalanced bargaining power between male and female in a relationship (males tend to dictate), this function is segregated by gender, with $\epsilon_{STI,female} > \epsilon_{STI,male}$.

We also model a saturation of sexual acts based on the number of concurrent partners. This is to avoid that an individual with many concurrent partners has an unrealistic rate of sex acts.

$$h_{nPartn}(n) = \frac{2}{1 + e^{-cn}} - 1$$

with c a saturation parameter.

5.2 Distribution of sex acts among partner types

Among these N sex acts, N_s, N_c and N_w were made with the male's spouse(s), casual partner(s) and sex worker ($N = N_s + N_c + N_w$). The distribution between these three categories is assumed to follow a multinomial law:

$$(N_s, N_c, N_w) \sim \text{Multinom}(N, \mathbf{p})$$

with $\mathbf{p} = (p_s, p_c, p_w)$ the probability vector defining the probabilities that a sex act will be with a spouse, a casual partner or a sex worker. The constraint is: $p_s + p_c + p_w = 1$.

The probability to engage with sex worker, it is based on the male's risk group r:

$$p_w = w_1 e^{-w_2(r^* - r)}$$

with r^* the highest risk group, w_1 and w_2 shape parameters.

The probability to have a sex act with a spouse is set to:

$$p_s = \alpha_s \frac{n_s}{n_s + n_c} \mathbf{1}_{n_c > 0} + (1 - p_w) \mathbf{1}_{n_c = 0}$$

with n_s (resp. n_c) the total number of spouses (resp. casual partners); parameter α a weighting factor depending on the average ages, average STI/HIV infections and average number of children among spouses and casual partnerships.

A constraint on α_s is $0 < \alpha_s <$ (# spouses/total # partnerships) such that $0 \leq p_s \leq 1$.

Finally, we have implicitly

$$p_c = 1 - p_s - p_w$$

The parameters of these probabilities will be calibrated on published data and surveys (DHS).

5.3 Distributing the number of sex acts between partners

Assume the male has k_s spouses. We distribute N_s acts between k_s females recursively with a binomial law. If $N_s^{(i)}$ is the number of sex acts allocated to the ith spouse, for $i \in \{1, ..., k_s\}$:

$$\left(N_s^{(1)}, ..., N_s^{(k_s)}\right) \sim \text{Multinom}\left(N_s, \frac{1}{k_s}\right)$$

Hence, there is no preference among spouses.

Similarly, the N_c sex acts with k_c casual partners are distributed with the same recursive formula, for $i \in \{1, ..., k_c\}$:

$$\left(N_c^{(1)}, ..., N_c^{(k_c)}\right) \sim \text{Multinom}\left(N_c, \frac{1}{k_c}\right)$$

5.4 Distributing sex acts types

Once the number of sex acts are allocated to each partner, the type of sex act must be specified. Sex act types allocation is first described for spouses, casual partners and sex workers will have the same methodology.

The $N_s^{(i)}$ sexual intercourses with his i^{th} spouse are distributed among the three sex act types (with condom, no condom low risk, no condom high risk). The number of sex acts performed with a condom is $N_{s,0}^{(i)}$, without condom and low risk practices $N_{s,1}^{(i)}$ and without condom and high risk practices $N_{s,2}^{(i)}$

$$\left(N_{s,0}^{(i)}, N_{s,1}^{(i)}, N_{s,2}^{(i)}\right) \sim \text{Multinom}(N_s^{(i)}; \mathbf{p_s})$$

with $\mathbf{p_s} = (p_{s,0}\,, p_{s,1}\,, p_{s,2})$ the vector of probabilities to engage in the respective sex act types.

The following functional forms are assumed for the probabilities:

$$p_{s,0} = p_{s,0}(r_m, r_f) = t_1 e^{-t_2(r_m+r_f)/r^*}$$

with t_1, t_2 shape parameters, r_f (resp. r_m) the risk group of the female (resp. male) and r^* the maximum risk group.

It is assumed that among those sex acts that are not performed with a condom, a fixed proportion β_s of the remaining will engage in medium-risk practices without condoms:

$$p_{s,1} = \beta_s(1 - p_{s,0})$$

Finally, implicitly we have:

$$p_{s,2} = 1 - p_{s,0} - p_{s,1}$$

Similarly, we have for casual partners:

$$\left(N_{c,0}^{(i)}, N_{c,1}^{(i)}, N_{c,2}^{(i)}\right) \sim \text{Multinom}(N_c^{(i)};\ \mathbf{p_c})$$

with $\mathbf{p_c}$ calculated the same way as $\mathbf{p_s}$.

5.5 Limits on the number of sex acts for females

Because the number of sex acts (within partnerships) is driven by males, there is a risk a female has a total number of sex acts, noted N_f here, unrealistically high if she has several partnerships.

Hence, a maximum rate of sexual intercourses for females is assumed, and noted $R_{\text{sex}}^{max,f}$. For a given period dt, if the number of sex acts allocated to a female is higher than $R_{\text{sex}}^{max,f}dt$, then the following corrective algorithm is applied to the number of sex acts for *both* female and male (noted N_m here) in this partnership:

If $N_f > R_{\text{sex}}^{max,f}dt$ then :

1. $N_f \leftarrow \min\left(N_f\,; \text{int}[R_{\text{sex}}^{max,f}dt]\right)$
2. $N_m \leftarrow \max\left(N_m - (N_f^{old} - N_f^{new}); 0\right)$

where int$[x]$ denotes the integer part of any real number x.

Note: This algorithm will be improved in a future version.

5.6 Sex acts of males with no partnership

Males with no partnership are simulated with a slightly different process as their sexual acts can only be with sexual workers, hence the distribution of sex acts between different partners is not relevant.

The number of sex acts is still assumed to follow a Poisson distribution

$$N \sim \text{Poisson}(R_{\text{sex}}^{single}\, dt)$$

But, the factors determining the effective rate from the maximum rate are different.

$$R_{\text{sex}}^{single} = R_{\text{sex}}^{max} \times R_M^{single} \times R_{cost}$$

with

$$R_M^{single} = h_{age}(A_m)\, h_{risk}(r)\, h_{STI}(s)\, h_{HIV}(\tau)$$

Compared to R_M for males in partnerships, the factor related to the number of partners is removed, as the male is assumed to have access to an ever-sufficient services from sex workers (economic costs aside).

A new factor representing the transaction cost of sex work services is introduced, R_{cost}. For simplicity, it is set to a constant. Its aim is to limit the number of visits to CSW.

6 Commercial sex workers

6.1 Recruitment

It is assumed commercial sex workers (CSW) are recruited in the population at a rate proportional to the population size. If $R^{\star}_{csw}$ is the maximum rate of recruitment and N the total population size, the number of CSW recruited during the period of time dt is distributed with a Poisson distribution:

$$N_{csw}^{\text{new}} \sim \text{Poisson}\left(R_{csw}\, N\, dt\right)$$

with

$$R_{csw} = \frac{1 + e^{-ab}}{1 + e^{a(x-b)}} R^{\star}_{csw}$$

where x is the proportion of the number of CSW to the total population and a and b two constants. The multiplicative logistic term is introduced to translate a saturation of the demand for CSW: recruitment tends to 0 as the proportion of CSW in the population grows.

The age of the newly recruited CSW is uniformly distributed between a pre-specified age range (e.g. 15 to 40 years old).

The infection status with respect to each STI is also set stochastically. We denote A_s the number of a newly recruited CSW infected with STI s. We assume that

$$A_s \sim \text{Binomial}(N_{csw}^{\text{new}}, p_s)$$

where p_s is the current population prevalence of the associated STI (s). The A_s individuals are picked randomly among the new N_{csw}^{new} CSWs. A new CSWs who has been (stochastically) infected, is assumed to have just contracted the infection (STI duration is set to 1 day).

Previous number of partner is arbitrarily set to Poisson((age-minsexage)/2) and the widow prevalence is set at the same level as the general population.

6.2 Cessation

Among all the current CSW in the population (N_{csw}), we assume the rate of individuals dropping out of commercial sex (q_{csw}) is proportional to their number.

$$N_{csw}^{\text{quit}} \sim \text{Binomial}\left(q_{csw}\, dt, N_{csw}\right)$$

The risk group of the quitting CSWs is assigned randomly (multinomial among all risk groups). Note that only the risk group (set at a distinctive high value) identifies a CSW from the rest of the population.

7 Disease transmission

The transmission of STI will be determined by the probability of transmission per sex act. This probability is calculated from an infectivity curve associated to the infected partner and a susceptibility factor associated to the susceptible partner.

7.1 Infectivity curve and susceptibility factor

Infectivity curve

Individuals infected with an STI have an infectivity curve associated to this infection, noted IC. The infectivity curve is normalized such that the peak(s) of infectiousness is 1. At time $t = 0$, the pathogen invade the individual and by definition $IC(0) = 0$.

The shape of this curve depends on the disease. A proxy for the shape of the infectivity curve is the viral load in genital secretions. Other features from the infected individual (age, co-infections, etc) can impact the shape of this curve. Detailed formulation of the infectivity curves for each STI is described in 7.3.

Susceptibility factor

The susceptible individual who is at risk of transmission during the sex act considered, has a specific susceptibility factor to a given STI, noted SF. Susceptibility is maximal when $SF = 1$. Let's assume the STI considered is the ith in the list of all STIs modelled. This factor is reduced with respect to circumcision status (for male only):

$$SF_i = SF_i^{\text{circum}}$$

where $SF_i^{\text{circum}} \leq 1$ are estimated from the literature.

Note: As default value for the whole population, no male is circumcised.

7.2 Probability of transmission

For one given sexual intercourse, the probability of transmission, PT, is calculated from both the infectivity curve and the susceptibility factor of the pair of individuals considered.

The type of sex act (with or without condom, low or high risk) also impacts the probability and is represented in a functional form with a range between 0 and 1, noted $SAT(type)$ for Sex Act Type. Because only 3 sex act types are considered, the domain of this function is $\{0, 1, 2\}$ with 2 representing high-risk sex (anal, dry-sex), 1 standard sex act and 0 sex act with condom. We have $SAT(2) = 1$ as no risk reduction is allowed when the riskiest sex act is performed, and $SAT(0)$ should be a tiny number to reflect the dramatic reduction of transmission risk when a condom is used. Because of the difference between STIs, the value of $SAT(1)$ is STI-specific.

It is also assumed there is a maximum probability of transmission per sex act for a given STI s, and is noted $PT^*_{0,s}$. This probability assumes no other STI co-infection.

Hence, the formula defining the probability of transmission for a given STI s, *without any other STI co-infections* is:

$$PT_{0,s}(t, I_1, I_2, type) = IC_s(t, I_1) \times SF_s(I_2) \times SAT_s(type) \times PT^*_{0,s}$$

with t the duration since infection of the infected partner, I_1 (resp. I_2) vector of relevant features (e.g. age) of the infectious (resp. susceptible) individual, and *type* the sex act type.

If the susceptible partner is already infected with another STI, then the transmission probability is increased. Given an odds-ratio C_{ij} for increased susceptibility to STI i when already infected with STI j, we assume the overall odds-ratio is

$$R_i = \max_j (C_{ij})$$

The susceptibility factor due to STI co-infections is assumed constant for a given pair of STIs and represented by a matrix C, where the columns represent the STI already infecting the individual (j) and the row the STI the individual is still susceptible to. Entries of the matrix C can be calibrated on published literature (as it is likely that co-infection increases susceptibility we have $1 \leq C_{ij}$).

Hence, the transmission probability taking into account any other STI co-infection is:

$$PT_s(t, I_1, I_2, type) = \frac{R_s \, PT_{0,s}}{1 + (R_s - 1)PT_{0,s}}$$

(formula implied from the odds-ratio definition $OR = \frac{p/(1-p)}{p'/(1-p')}$)

For a pair of individuals who has n_y sex acts of type y during a given period, the probability of transmission of a given STI s after these multiple sex acts is noted MPT and is given by the following formula:

$$MPT_s(t, I_1, I_2) = 1 - \prod_{y=0}^{2} [1 - PT_s(t, I_1, I_2, y)]^{n_y}$$

7.3 Probabilities of transmission for every STI

Here, the infectivity curves and susceptible factors are defined for every STI modelled.

7.3.1 HIV

The infectivity curve of HIV is defined by pieces to represent the different stages of the natural history of HIV. The parameters used for its definition are summarized in Table 1

Table 1: Parameters for the infectivity curve of HIV

Notation	Interpretation
Tvl^*_{HIV}	Time after initial infection when viral load peaks
q_{HIV}	Shape parameter of acute infection
σ_{HIV}	Dispersion of the duration of acute phase
$VL_{chronic}$	Fraction of peak viral load when chronic stage starts
$D_{chronic}$	Duration (in years) of the chronic infectious stage
$r_{chronic}$	Rate of viral load progression during the chronic stage
D_{AIDS}	Duration (in years) of AIDS (death as end-point)

The infectiousness during the acute period following initial infection is represented by (the subscript HIV is dropped for readability):

$$IC_{HIV,acute}(t) = \exp\left(-\frac{(t - Tvl^*)^{2q}}{\sigma^{2q}}\right)$$

with $0 < t < T_c$ the time since initial infection and $T_c = \sigma(-\ln(VL_{chronic}))^{1/2q} + Tvl^*$, the time after initial infection when the chronic stage starts.

For the chronic phase for $T_c \leq t < T_c + D_{chronic}$

$$IC_{HIV,chronic}(t) = VL_{chronic}\, e^{(t-T_c)r_{chronic}}$$

And finally for the AIDS stage, for $t \geq T_{AIDS} = T_c + D_{chronic}$

$$IC_{HIV,AIDS}(t) = IC_{HIV,chronic}(T_{AIDS})e^{-(t-T_{AIDS})\ln(VL_{chronic})/D_{AIDS}}$$

The end-point being death, and it is assumed the infectivity level is back at its peak value at this time.

The infectivity curve of HIV *without any other co-infections* is given by putting together the three stages:

$$IC_{HIV only}(t) = IC_{HIV,acute}(t) + IC_{HIV,chronic}(t) + IC_{HIV,AIDS}(t)$$

When the infected individual is co-infected with another STI, the HIV infectiousness is assumed to increase and mirror the other STI infectiousness, up to a given ratio.

$$IC_{HIV,coSTI}(t) = \left(\sum_s RI_{coSTI}(s) IC_s(t)\right) IC_{HIV only}(t)$$

with s summing on all STI modelled and $RI_{coSTI}(s)$ is the rebound of HIV infectivity due to co-infection with STI s.

Finally, the full infectivity curve for HIV is

$$IC_{HIV}(t) = IC_{HIV only}(t) + IC_{HIV,coSTI}(t)$$

Practically, the value is capped at one, that is $IC_{HIV}(t) = \min(1, IC_{HIV only}(t) + IC_{HIV,coSTI}(t))$.

It is implicitly assumed that co-infections cannot increase HIV infectivity beyond the peak infectivity when only infected with HIV. It is also assumed that the increase of HIV infectivity mirrors the pattern of infectivity of the co-occurring STI.

7.3.2 Syphilis (Treponema pallidum, Tp)

Primary syphilis: After initial exposure, a primary chancre develops at the site of entry (usually genital) after 3-90 days (average 3 weeks) [4, 5]. It takes about 4-6 weeks for spontaneous resolution (without treatment) of the primary chancre.

Secondary syphilis: Up to 85% of cases will progress to generalized lesions[5], within 4-10 weeks after the appearance of the initial chancre[4, 5]. A small proportion of cases, 10%[5] 5-22%[4], will develop highly infectious chancres (condylomata lata). Spontaneaous resolution occurs within less than 3 months[5] or several weeks[4].

Early latent syphilis: About 25% of cases experiences a recurrence of secondary syphilis symptoms during a window period of about 6 months

Syphilis (untreated) is expected to be sexually transmissible during 2 years after initial infection[5]. Late latent and tertiary phases are not infectious. Tertiary phase occurs 15-30 years later and is associated with increased mortality in some cases.

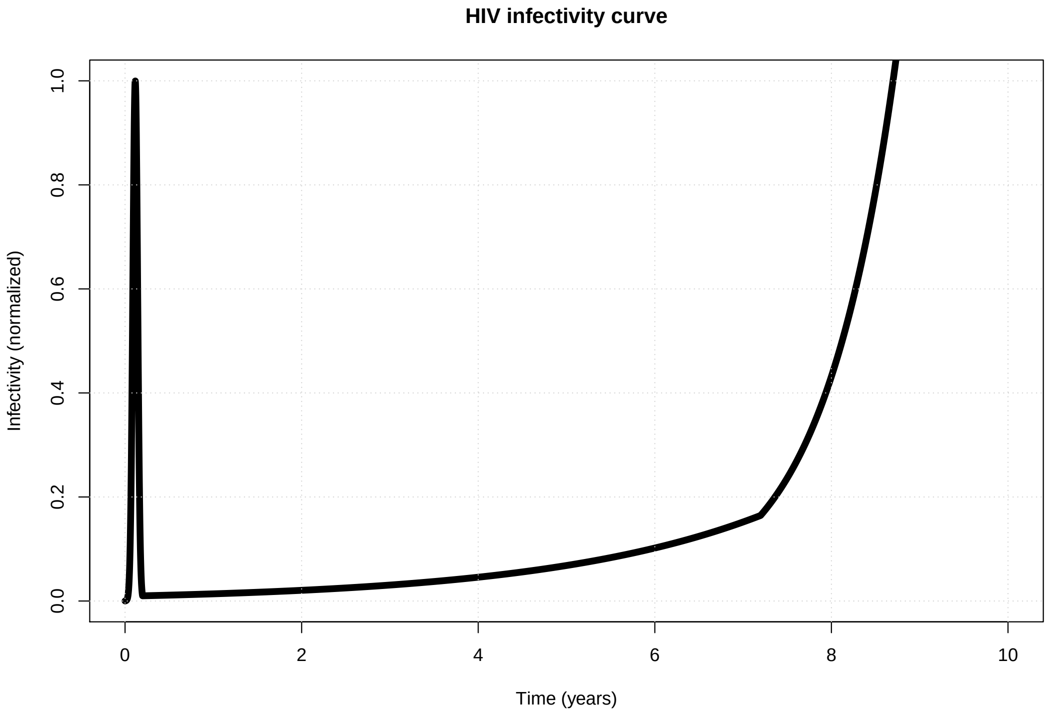

Figure 3: Infectivity curves for HIV.

Co-infection with HIV might be associated with a higher Tp virulence, but syphilis treatment is the same as HIV-uninfected patients[4]. The infectivity curve is defined with respect to the syphilitic stages.

Primary syphilis infectivity curve is represented with the pseudo-beta function defined in appendix 11.1:

$$IC_{Tp.1}(t) = v_{Tp.1} \times \mathcal{B}\left(\frac{(t - L_{Tp.1})^+}{D_{Tp.1}}, a_{Tp.1}, b_{Tp.1}\right)$$

with $a_{Tp.1}$ and $b_{Tp.1}$ shape parameters, $L_{Tp.1}$ the latent period before being infectious (suggested 30 days), $D_{Tp.1}$ the infectiousness duration of primary syphilis (suggested 5 weeks) and $v_{Tp.1}$ the relative virulence of this primary stage (suggested 0.7) compared to peak infectivity (when the value of the infectivity curve is 1).

Secondary syphilis is defined similarly, but with two possibilities for the infectivity curve reflecting the fact that some cases will develop highly infectious condylomata lata.

In the absence of condylomata:

$$IC_{Tp.2}^{no\,condy}(t) = v_{Tp.2} \times \mathcal{B}\left(\frac{(t - L_{Tp.2})^+}{D_{Tp.2}}, a_2, b_2\right)$$

when condylomata develop:

$$IC_{Tp.2}^{condy}(t) = v_{Tp.2}^{condy} \times \mathcal{B}\left(\frac{(t - L_{Tp.2})^+}{D_{Tp.2}^{condy}}, a_2^{condy}, b_2^{condy}\right)$$

with a_2 and b_2 shape parameters, $L_{Tp.2}$ the latent period from infection before secondary syphilis is triggered(suggested L_{Tp} plus 7 weeks), $D_{Tp.2}$ the infectiousness duration of secondary syphilis

(suggested 8 weeks) and $v_{Tp.2}$ the relative virulence of this secondary stage (suggested 0.7) compared to peak infectivity.

The parameters with the superscript *condy* apply to the case when condylomata develop. In this case, the parameters are assumed to be different.

The probability that condolymata develop is represented by the binary random variable κ_{condy} that takes value 1 with probability p_{condy} (suggested 0.15), else is 0.

Hence, for secondary syphilis we have:

$$IC_{Tp.2}(t) = \kappa_{condy} IC_{Tp.2}^{condy}(t) + (1 - \kappa_{condy}) IC_{Tp.2}^{no\,condy}(t)$$

Early latent syphilis is considered as a repeat of symptoms that occurred during secondary syphilis, hence it is defined similarly:

$$IC_{Tp.el}(t) = v_{Tp.el} \times \mathcal{B}\left(\frac{(t - L_{Tp.el})^+}{D_{Tp.el}}, a_{el}, b_1 el\right)$$

with a_{el} and b_{el} shape parameters, $L_{Tp.el}$ the latent period before earl latent stage is triggered (suggested L_{Tp} plus 12 months), $D_{Tp.1}$ the infectiousness duration of primary syphilis (suggested 5 weeks) and $v_{Tp.el}$ the relative virulence of this primary stage compared to peak infectivity.

Finally, the total infectivity curve for syphilis is:

$$IC_{Tp.el}(t) = IC_{Tp.1}(t) + \kappa_{Tp.2} IC_{Tp.2}(t) + \kappa_{Tp.el} IC_{Tp.el}(t)$$

with $\kappa_{Tp.2}$ (resp. $\kappa_{Tp.el}$) the binary random variable taking value 1 with probability $p_{Tp.2}$ (resp. $\kappa_{Tp.el)}$ representing the probability to develop secondary (resp. early latent) syphilis. (suggested: $p_{Tp.2} = 0.85$ and $\kappa_{Tp.el} = 0.20$)

A graphical representation is given in Figure 4

7.4 Mother-to-child (vertical) transmission

When a female is both pregnant and infected with a STI, transmission of the pathogen to the children is modeled as a stochastic event. For all STI except syphilis, the probability of mother-to-child transmission is assumed constant (it does not depend on pregnancy stage and duration of infection):

$$p_{MTCT} = \textbf{constant}$$

For syphilis, there is some evidence [6, 7] that risk of vertical transmission is higher during the early stages of syphilis infection. Hence, a decreasing logistic shape is assumed for the probability of syphilis mother-to-child transmission:

$$p_{MTCT}^{Tp} = \frac{a}{1 + e^{b(\tau - c)}}$$

with τ the duration of syphilis infection and a, b, c shape parameters.

For every pregnant female infected with an STI, the vertical transmission to the new born is decided by drawing a random variable from a Bernoulli distribution with probability p_{MTCT}.

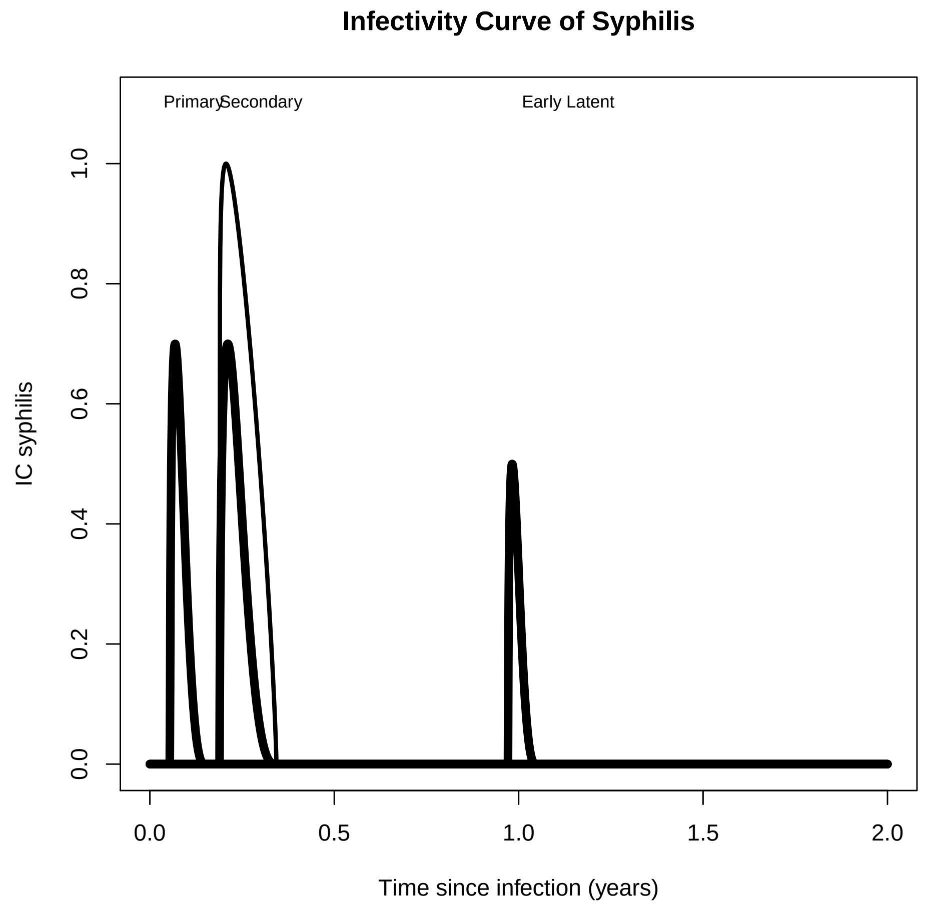

Figure 4: Infectivity curve for Syphilis. The thin curve in the secondary syphilis stage represents the case when highly infectious condolymata develop.

8 Treatment and vaccination

8.1 Treatment implementation

When an individual is infected with an STI, receiving a treatment will affect (most likely reduce) her/his infectiousness, symptomatic status and increase the chances of being cleared from the pathogen, if the STI is curable.

An individual starting a treatment will go through several steps before potentially having a positive outcome.

8.1.1 Treatment microbiological failure

There is a risk of failure with any treatment. An individual may poorly respond to prescribed drugs, or be infected with a drug-resistant strain of the pathogen (adherence is treated separately hereafter).

For a given STI, let TMS be the random variable representing microbiological treatment success (conditional on full adherence) and p_{fail} the probability of treatment failure. It is assumed that

TMS has a Bernoulli distribution ($TMS = 1$ is successful treatment):

$$TMS \sim \text{Bern}(1 - p_{fail})$$

8.1.2 Adherence

Right from treatment inception, adherence is determined based on the individual's risk group and symptomatic status. A non-adherent behaviour will reduce the amount of drug intake. If adherence A is measured as the fraction of the optimal drug intake ($0 \leq A \leq 1$, with $A = 1$ being full adherence), then it is assumed

$$A = a_0 e^{-a_1 r} a_2$$

with r the risk group of the individual, a_0 the maximum adherence, a_2 a reducing factor when the infection is asymptomatic (it is assumed that a symptomatic infection motivates more to adhere to treatment).

8.1.3 Treatment reduction effect

Treatment is affecting the infectivity curve, with the aim of reducing it to either 0 for curable STI or a very small value for non-curable STIs (e.g. HIV).

We assume there is an hypothetical treatment reduction effect (TRE^*) conditional on microbiological success and full adherence. Duration of treatment has an optimal length note TD^*. TRE is a function of treatment duration τ and we have $TRE^*(0) = 1$ (no reduction of infectiousness at the very start of treatment) and $TRE^*(TD^*) = 0$ or ϵ (treatment has cured [when STI is curable] or heavily suppressed [when non-curable] the pathogen). The actual treatment reduction effect TRE is given by

$$TRE(A, TMS, \tau) = [A \times TRE^*(\tau) + (1 - A)]\, TMS + (1 - TMS)$$

Function TRE^* will be defined specifically for each STI.

The infectivity curve before treatment (IC) is thus modified into an infectivity curve during treatment (IC^{treat}):

$$IC^{treat}(t, \tau) = IC(t) \times TRE(A, TMS, \tau)$$

For curable STIs, cure is achieved by assessing the value of a random variable with a Bernoulli distribution

$$Cure \sim \text{Bern}(TRE(TD^*))$$

If $Cure = 1$, the STI is cured. If $Cure = 0$ the infectivity curve is set back, as if $TRE = 1$.

8.2 Vaccine implementation

The individuals eligible for vaccination are selected according to pre-defined criteria defined at the simulation level (for example age). Given an individual receives a vaccine injection, his/her immunity to the STI is instantaneously set to 100% with probability $1 - p_{fail}$, with p_{fail} the failure probability of the vaccine. The immunity decreases at the exponential rate w, such that immunity t time units after (successful) vaccination is:

$$\text{immunity}(t) = e^{-wt}$$

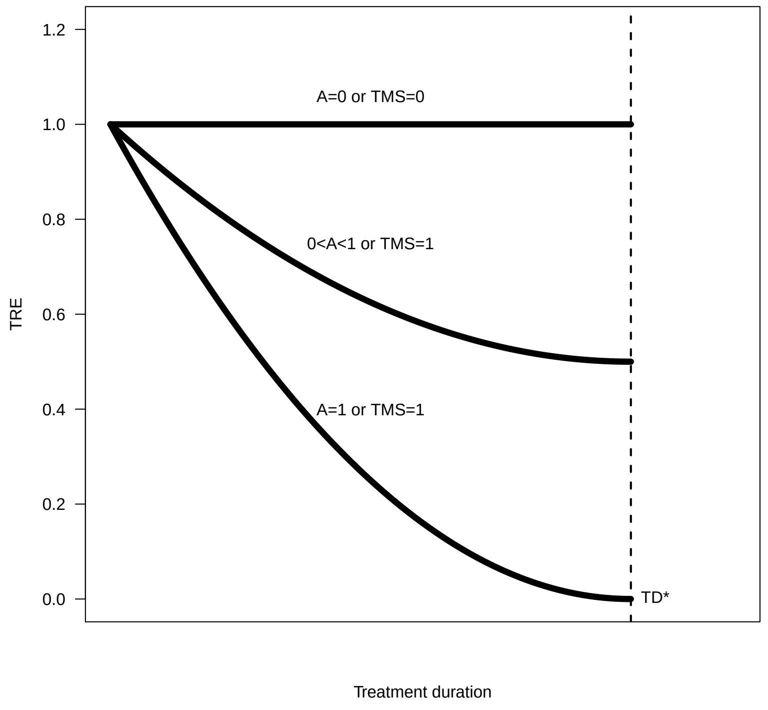

Figure 5: Treatment reduction effect.

The susceptibility factor to that STI at time t, $SF(t)$, is updated every time step:

$$SF(t) = SF(t-1)(1 - \text{immunity}(t))$$

A vaccinated individual can acquire the associated STI if the vaccine effectiveness has waned sufficiently. In that cases, the model gives the possibility to simulate a reduction of infectiousness provided by the previous vaccination. Put simply, the vaccine is not strong enough to protect the individual from infection, but if infection happens, the individual will be less infectious. The vaccine reduction effect (VRE) on infectiousness is simply modelled as a constant multiplicative factor applied to the infectivity curve.

9 Calibration

This agent-based model has many model parameters, that are all summarized in Tables 2, 3, 4, 5, 6 and 7. They can be classified in groups that specifically affect:

- demographics
- partnerships dynamics
- sexual behaviour
- diseases natural history
- treatments

Only the parameters in the "diseases natural history" and "treatments" were not calibrated to data. Fixed values were assumed either based on the literature or arbitrarily set (Tables 5, 6, 7).

The fitting procedure consist in fitting groups of parameters sequentially. Many "feedback loops" exist: for example, demographics can affect indirectly STI prevalence (for example, a lack of young individuals could force disease transmission to stay in older cohorts, limiting te prevalence), but STI prevalence can also affect demographics (e.g. HIV-induced deaths). Hence, given the large number of parameters, a practical way to identify parameters is to fit them sequentially by groups. The order of this sequence is arbitrary and justified as follows. Demographic processes are considered the most fundamental, hence they should be fitted first. Then partnership dynamics and sexual behaviour are fitted to relevant data.

Demographic parameters were first fitted. Not all parameters were fitted: crude birth rate and children mortalities were directly inputed from the literature. Only the deaths hazard were fitted and the fitting procedure was relatively straightforward (simply matching a pre-specified life expectancy with or without HIV). These demographic parameters remained unchanged during the next fitting steps. See Table 2.

The partnerships parameters were i) set to a fixed value taken either from the literature or arbitrarily, ii) fitted to the DHS database, or iii) fitted to prespecified STI prevalences (Table 3). Similarly, sexual behaviour parameters were i) set to a fixed value or ii) fitted to prespecified STI prevalences (Table 4).

The calibration method used is an Approximate Bayesian Approximation (ABC). The summary statistic F is simply the sum of squared differences to the target data. STI prevalences are fitted by risk groups and ages. We have:

$$\begin{aligned} F(X) &= (PartnerRatio(X)/target_{PR} - 1)^2 \\ &\quad + (MedAgeMar(X)/target_{MAM} - 1)^2 \\ &\quad + \sum_s (STIRiskGrpPrev_s(X)/target_{RGP,s} - 1)^2 \\ &\quad + \sum_{a,s} (STIAgePrev_{a,s}(X)/target_{AP,a,s} - 1)^2 \end{aligned}$$

with $target_x$ the target values from the data, $PartnerRatio$ the proportion of individuals in partnerships from the simulation (fitted on DHS), $MedAgeMar$ the median age at marriage for women (fitted on DHS), $STIRiskGrpPrev_s$ the prevalence of each STI by risk group (no data, scenario assumptions) and $STIAgePrev_s$ the STI prevalence by age (shape for HIV fitted on DHS, levels are scenario assumptions).

10 Simulations

Below is the high-level algorithm used to perform epidemiological analyses.

1. start with initial population, no partnerships, no STI
2. run simulation long enough such that the partnership dynamics are in a steady state. Fit of equilibrium values to target demographic and partnership data should be performed at that step
3. introduce STIs into the population and run long enough such that prevalence and incidence of all STIs reach their steady state
4. determine a date after the prevalence and incidence steady states are reached to implement an intervention (e.g., vaccine)
5. set a horizon where the baseline (i.e. no intervention) and intervention simulations are compared

11 Appendix

11.1 Pseudo-beta shape function

For infectivity curves associated with STIs of limited duration, the shape function was inspired from the beta distribution density (because it has a bounded support).

$$\mathcal{B}(x,a,b) = x^{a-1}(1-x)^{b-1}/C$$

where C is the normalizing constant such that the maximum value of $\mathcal{B}$ is 1 on the interval [0;1]:

$$C = \left(\frac{a-1}{a+b-2}\right)^{a-1}\left(\frac{b-1}{a+b-2}\right)^{b-1}$$

It is assumed that for any $a, b > 1$ and $x < 0$ and $x > 1$ we have $\mathcal{B}(x,a,b) = 0$. The maximum of $\mathcal{B}$ is reached at $x_{max} = (a-1)/(a+b-2)$.

11.2 Tables of all parameters

Parameter	Description	Value	Source
`birthRate`	Annual crude birth rate for the whole population	0.037	The World Bank (SSA only)
m_{infant}	Annual infant(< 1 year-old) mortality rate	0.06	CIA World Fact Book (data averaged and rounded to the nearest percentage for SSA)
m_{child}	Annual children(between 1 and 5 year-old) mortality rate	0.04	The World Bank (SSA only)
k	shape parameter for Weibull distribution of death hazard	2.5	fitted to average life expectancy of 70 years
λ	scale parameter for Weibull distribution of death hazard	0.01	fitted to average life expectancy of 70 years
k'	shape parameter for Weibull distribution of death hazard after acquiring HIV	12.98	fitted to maximum survival of 10 years after HIV acquisition
λ'	scale parameter for Weibull distribution of death hazard after acquiring HIV	0.05	fitted to maximum survival of 10 years after HIV acquisition
`minSexAge`	Minimum age (in years) for sexual contact	12	assumption
`maxSexAge`	Maximum age (in years) for sexual contact	60	assumption
`minCSWAge`	Minimum age (in years) for a female to practice commercial sex work	15	assumption
`maxCSWAge`	Maximum age (in years) for a female to practice commercial sex work	45	assumption

Table 2: Exhaustive list of all model parameters related to demographics. These parameters are the same across all synthetic populations A, B and C. SSA: Sub-Saharan African countries

Parameter	Description	Value(s)	Source
**Formation**			
r_f^*	maximum formation rate (female dominance)	2.0 ; 3.1 ; 3.1	fitted to target STIs prevalence
s_{age}	shape parameter for age component of partnership formation	0.27	fitted to target STIs prevalence
a^*	location parameter for the age component of partnership formation	43 years	assumption
g_{min}	minimum age gap (male age minus female age) for partnership formation	-7 years	assumption
$\bar{g}$	mean age gap at partnership formation	10.1 years	fitted to target STIs prevalence
s_{gap_1}	shape parameter for age gap component in partnership formation	5	assumption
s_{gap_2}	shape parameter for age gap component in partnership formation	2	assumption
s_0^{risk}	shape parameter for risk group component in partnership formation	0.3	fitted to target STIs prevalence
s_1^{risk}	shape parameter for risk group component in partnership formation	1.0	fitted to target STIs prevalence
q	shape parameter for partner deficit component in partnership formation	0.40	fitted to target STIs prevalence
$a_{sympt,f}$	probability component of partnership formation if female is symptomatic from any STI	0.82	fitted to target STIs prevalence
$a_{sympt,m}$	probability component of partnership formation if male is symptomatic from any STI	$a_{sympt,f}$	n/a
**Spousal progress**			
sp^*	maximum annual rate of spousal progression	0.05	based on DHS (proportion living in spousal union)
$\bar{A}_f$	mean female age progressing to spousal union	18 years	based on DHS (Women's median age at first marriage)
σ_{A_f}	standard deviation of female age progressing to spousal union	9.25 years	assumption
$\bar{G}$	mean age gap for a partnership progressing to spousal union	4.75 years	assumption based on [8]
σ_G	standard deviation of age gap for a partnership progressing to spousal union	14.2 years	assumption based on [8]
k_1	mean partnership duration when progression to spousal union occurs	5 years	assumption
k_2	variance of partnership duration when progression to spousal union occurs	3 years	assumption
$\bar{\Delta}$	mean age gap difference b/w youngest existing wife and candidate wife	12 years	assumption
σ_Δ	standard deviation of age gap difference b/w youngest existing wife and candidate wife	5 years	assumption
**Dissolution**			
δ^*	maximum annual dissolution rate	0.1 ; 0.4 ; 0.4	fitted to target STIs prevalence
ϵ	probability component for dissolution of a spousal partnership	0.06	based on DHS (proportion living in spousal union)
dur_1	shape parameter (duration) of probability component for dissolution	0.324	fitted to target STIs prevalence
dur_2	shape parameter (duration) of probability component for dissolution	0.69	fitted to target STIs prevalence
dur_3	shape parameter (duration) of probability component for dissolution	0.15	fitted to target STIs prevalence
drisk_1	shape parameter (risk group) of probability component for dissolution	0.7	fitted to target STIs prevalence
dage_1	shape parameter (partners' age) of probability component for dissolution	75.9 years	fitted to target STIs prevalence
dage_2	shape parameter (partners' age) of probability component for dissolution	0.1	fitted to target STIs prevalence
dage_{min}	shape parameter (partners' age) of probability component for dissolution	0.7	fitted to target STIs prevalence
q	shape parameter for partner deficit component in partnership dissolution	2.1	fitted to target STIs prevalence
q_{min}	shape parameter for partner deficit component in partnership dissolution	0.3	fitted to target STIs prevalence
$d_{symptom}$	dissolution probability relative reduction for asymptomatic case in partnership	0.55	fitted to target STIs prevalence

Table 3: Exhaustive list of all model parameters related to partnerships. A single value means it is shared for all synthetic populations. Several values indicates the one associated to each synthetic populations A, B and C respectively. DHS data were averaged across the following sub-Sahara African countries (DHS recode version in parentheses): Burkina-Faso (4), Cameroon (4), Ethiopia (6), Kenya (5), Lesotho (5), Malawi (5), Rwanda (6), Senegal (6), Swaziland (5), Zambia (5), Zimbabwe (6).

Parameter	Description	Value(s)	Source
`propRisk0`	proportion of the population in risk group 0 (safest)	0.60 ; 0.25 ; 0.35	fitted to target STIs prevalence
`propRisk1`	proportion of the population in risk group 1	0.35 ; 0.55 ; 0.50	fitted to target STIs prevalence
`propRisk2`	proportion of the population in risk group 2 (riskiest)	0.05 ; 0.20 ; 0.15	fitted to target STIs prevalence
c_1	parameter for the geometric distribution for concurrency (female and male)	0.35	fitted to target STIs prevalence
$c_{2,f}$	parameter for the geometric distribution for concurrency (female)	0.6	fitted to target STIs prevalence
$c_{2,m}$	parameter for the geometric distribution for concurrency (male)	0.7	fitted to target STIs prevalence
R_{sex}^{max}	maximum annual sex acts rate for males	360	assumption
$R_{sex}^{max,f}$	maximum annual sex acts rate for females (non CSW)	500*	assumption
a_{peak}	shape parameter related to sexual activity peak age	20 years	assumption
σ_{age}	shape parameter related to standard deviation of sexual activity peak age	30 years	assumption
q	shape parameter related to frequency of age-dependant sexual activity	5.0	assumption
ϵ_{risk}	parameter related to frequency of risk-dependant sexual activity	0.4	assumption
ϵ_{STI}	frequency reduction of STI symptoms-dependant sexual activity	0.8	assumption
c	saturation parameter of sexual activity in concurrent partnerships	2.0	assumption
α_s	spouse preference parameter in multinomial distribution	2.0	assumption
w_1	shape parameter for the probability of risk-dependent sex acts with CSW	0.5	assumption
w_2	shape parameter for the probability of risk-dependent sex acts with CSW	8.0	assumption
R_{cost}	factor to limit the overall number of visits to CSW	0.1	assumption
t_1	shape parameter for the distribution of sex act types	0.2	assumption
t_2	shape parameter for the distribution of sex act types	0.7	assumption
β_s	proportion low versus high risk sex act types	0.76	fitted to target STIs prevalence
R_{csw}^{*}	maximum CSW annual recruitment rate	0.005	fitted to target STIs prevalence
a	shape parameter for CSW recruitment saturation	800	assumption
b	shape parameter for CSW recruitment saturation	0.0005	assumption
q_{csw}	CSW annual cessation rate	0.049	fitted to target STIs prevalence

Table 4: Exhaustive list of all model parameters related to sexual behaviour. A single value means it is shared for all synthetic populations. Several values indicates the one associated to each synthetic populations A, B and C respectively. CSW: commercial sex worker. *: The limit of sexual activity for females is set higher than the one for males. For the vast majority of the female population, this difference of maximum sexual activity has no impact on the simulations because the average sexual activity is well below that limit. But for the highest risk group females, that limit was sometimes reached in simulations. Hence, the sexual activity limit for all females was increased to a level that was less likely reached (e.g., 500). Note that because the sexual activity within partnerships is male-driven, increasing this upper limit from 360 to 500 – for females only – does not impact the rate of sexual activity.

Parameter	Description	Value	Source
**HIV**			
Tvl^*_{HIV}	Time after initial infection when viral load peaks	6 weeks	assumption based on [9]
q_{HIV}	Shape parameter of acute infection	2	assumption based on [9]
σ_{HIV}	Dispersion of the duration of acute phase	2 weeks	assumption based on [9]
$VL_{chronic}$	Fraction of peak viral load when chronic stage starts	10^{-2}	assumption based on [9]
$D_{chronic}$	Duration (in years) of the chronic infectious stage	7 years	assumption based on [9]
$r_{chronic}$	Rate of viral load progression during the chronic stage	0.4	assumption based on [9]
D_{AIDS}	Duration (in years) of AIDS (death as end-point)	1 year	assumption based on [9]
$PT^*_{0,HIV}$	maximum probability of HIV transmission per sex act	0.06	assumption based on [10]
$SAT_{HIV}(1)$	multiplicative factor reducing transmission probability for low risk sex type	0.1	assumption based on [11]
p^{HIV}_{MTCT}	HIV mother to child transmission probability	0.3	assumption based on [12]
**Syphilis**			
$v_{Tp.1}$	primary syphilis virulence (relative to peak)	0.7	assumption based on [4, 5]
$a_{Tp.1}$	shape parameter primary syphilis	1.05	assumption based on [4, 5]
$b_{Tp.1}$	shape parameter primary syphilis	2	assumption based on [4, 5]
$L_{Tp.1}$	latent period primary syphilis	4 weeks	assumption based on [4, 5]
$D_{Tp.1}$	infectiousness duration primary syphilis	6 weeks	assumption based on [4, 5]
$v_{Tp.2}$	secondary syphilis virulence (relative to peak)	0.7	assumption based on [4, 5]
$a_{Tp.2}$	shape parameter secondary syphilis	1.5	assumption based on [4, 5]
$b_{Tp.2}$	shape parameter secondary syphilis	4	assumption based on [4, 5]
$L_{Tp.2}$	latent period secondary syphilis	16 weeks	assumption based on [4, 5]
$D_{Tp.2}$	infectiousness duration secondary syphilis	10 weeks	assumption based on [4, 5]
p_{condy}	probability that condylomata develop in secondary syphilis	0.15	assumption based on [4, 5]
$v^{condy}_{Tp.2}$	secondary syphilis w/ condylomata virulence (relative to peak)	1.0	assumption based on [4, 5]
$a^{condy}_{Tp.2}$	shape parameter secondary syphilis w/ condylomata	1.1	assumption based on [4, 5]
$b^{condy}_{Tp.2}$	shape parameter secondary syphilis w/ condylomata	1.8	assumption based on [4, 5]
$D^{condy}_{Tp.2}$	infectiousness duration secondary syphilis w/ condylomata	10 weeks	assumption based on [4, 5]
$L_{Tp.el}$	latent period early latent syphilis	1 year	assumption based on [4, 5]
$D_{Tp.el}$	infectiousness duration early latent syphilis	8 weeks	assumption based on [4, 5]
$v_{Tp.el}$	secondary syphilis w/ condylomata virulence (relative to peak)	1.0	assumption based on [4, 5]
$a_{Tp.el}$	shape parameter early latent syphilis	1.5	assumption based on [4, 5]
$b_{Tp.el}$	shape parameter early latent syphilis	4	assumption based on [4, 5]
$D_{Tp.el}$	infectiousness duration early latent syphilis w/ condylomata	8 weeks	assumption based on [4, 5]
$PT^*_{0,Tp}$	maximum probability of syphilis transmission per sex act	0.45	assumption based on [4, 5]
$SAT_{Tp}(1)$	multiplicative factor reducing transmission probability for low risk sex type	0.9	assumption based on [4, 5]
p^{Tp}_{MTCT}	syphilis mother to child transmission probability	0.9	assumption based on [13]

Table 5: Exhaustive list of all model parameters related to HIV and syphilis

Description	Baseline Value	Source
HIV infectiousness rebound when syphilis co-infection (prop. peak infectivity)	0.5	assumption
Syphilis infectiousness rebound when HIV co-infection	0	assumption
Syphilis susceptibility increase when already infected with HIV	1.5	assumption
HIV susceptibility increase when already infected with syphilis	2.5	[14, 15, 16]

Table 6: Exhaustive list of all model parameters related to HIV/syphilis co-infection

Description	Baseline Value	Source
probability treatment failure for HIV	0.01	assumption
probability treatment failure for syphilis	0.0	assumption
maximum adherence (a_0)	0.98	assumption
adherence reduction factor when asymptomatic (a_2)	0.7	assumption
shape parameter for adherence reduction factorbased on risk group (a_1)	0.1	assumption
maximum adherence (a_0)	0.98	assumption

Table 7: Exhaustive list of all model parameters related to STI treatment

References

[1] R Core Team. R: A Language and Environment for Statistical Computing;.

[2] Dietz K, Hadeler K. Epidemiological models for sexually transmitted diseases. Journal of Mathematical Biology. 1988;26(1):1–25.

[3] Luke N, Kurz KM. Cross-generational and transactional sexual relations in sub-Saharan Africa. Washington, DC: International Center for Research on Women (ICRW). 2002;.

[4] Kent ME, Romanelli F. Reexamining Syphilis: An Update on Epidemiology, Clinical Manifestations, and Management. Annals of Pharmacotherapy. 2008 Jan;42(2):226–236.

[5] Horváth A. Biology and Natural History of Syphilis. In: Sexually Transmitted Infections and Sexually Transmitted Diseases. Berlin, Heidelberg: Springer Berlin Heidelberg; 2011. p. 129–141.

[6] Karumudi UR, Augenbraun M. Syphilis and HIV: a dangerous duo. Expert Review of Anti-infective Therapy. 2005 Oct;3(5):825–831.

[7] Karp G, Schlaeffer F, Jotkowitz A, Riesenberg K. Syphilis and HIV co-infection. European Journal of Internal Medicine. 2009 Jan;20(1):9–13.

[8] Barbieri M, Hertrich V, Grieve M. Age difference between spouses and contraceptive practice in sub-Saharan Africa. Population (english edition). 2005;60:617–654.

[9] CDC. Report of the NIH Panel to Define Principles of Therapy of HIV Infection and Guidelines for the Use of Antiretroviral Agents in HIV-Infected Adults and Adolescents. MMWR Morbidity and mortality weekly report. 1998 Apr;47:1–91.

[10] Boily MC, Baggaley RF, Wang L, Masse B, White RG, Hayes RJ, et al. Heterosexual risk of HIV-1 infection per sexual act: systematic review and meta-analysis of observational studies. The Lancet Infectious Diseases. 2009 Jan;9(2):118–129.

[11] Hughes JP, Baeten JM, de Bruyn G, Inambao M, Kilembe W, Farquhar C, et al. Determinants of Per-Coital-Act HIV-1 Infectivity Among African HIV-1-Serodiscordant Couples. The Journal of Infectious Diseases. 2012 Jan;205(3):358–365.

[12] De Cock KM, Fowler MG, Mercier E, de Vincenzi I. Prevention of mother-to-child HIV transmission in resource-poor countries: translating research into policy and practice. JAMA. 2000;.

[13] Berman SM. Maternal syphilis: pathophysiology and treatment. BULLETIN-WORLD HEALTH ORGANIZATION. 2004 Jun;82(6):433–438.

[14] Røttingen JA, Cameron DW, Garnett GP. A systematic review of the epidemiologic interactions between classic sexually transmitted diseases and HIV: how much really is known? Sexually transmitted diseases. 2001;28(10):579–597.

[15] Fleming DT, Wasserheit JN. From epidemiological synergy to public health policy and practice: the contribution of other sexually transmitted diseases to sexual transmission of HIV infection. Sexually transmitted infections. 1999;75(1):3–17.

[16] Sexton J, Garnett G, R ttingen JA. Metaanalysis and Metaregression in Interpreting Study Variability in the Impact of Sexually Transmitted Diseases on Susceptibility to HIV Infection. Sexually transmitted diseases. 2005 Jun;32(6):351–357.
