# Supplementary material for: Epidemiological impact of a syphilis vaccine: a simulation study
Source: Epidemiol Infect. 2016 Aug 1;144(15):3244–52. doi: 10.1017/S0950268816001643 (PMC5080673; doi:10.1017/S0950268816001643)
Supplement: Supplementary file 1 [file hygsup.zip › S0950268816001643sup003.pdf]

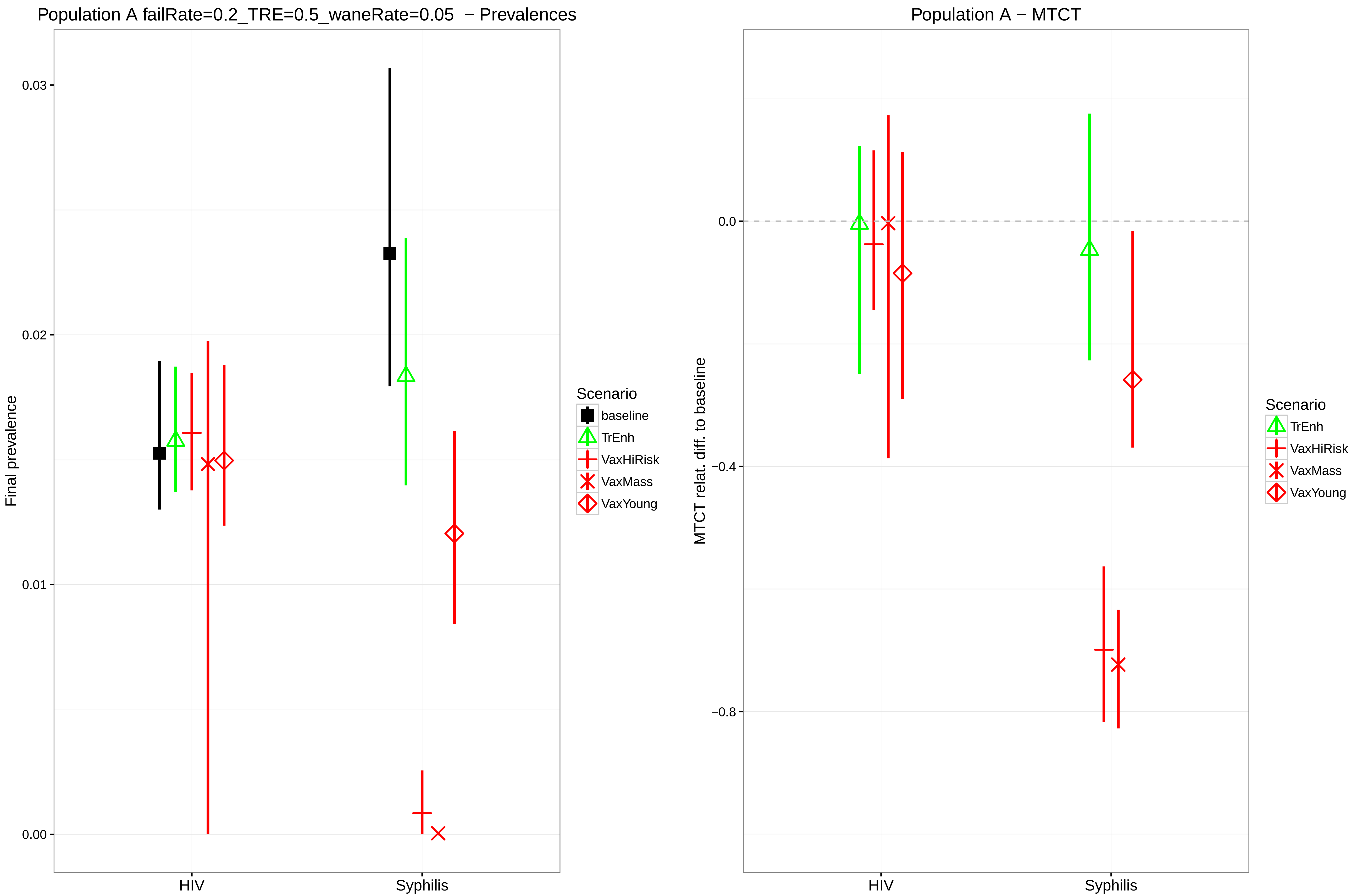

Population A failRate=0.2_TRE=0.5_waneRate=0.05 − Prevalences
Final prevalence
0.03
0.02
0.01
0.00
HIV
Syphilis
Scenario
baseline
TrEnh
VaxHiRisk
VaxMass
VaxYoung
Population A − MTCT
MTCT relat. diff. to baseline
0.0
−0.4
−0.8
HIV
Syphilis
Scenario
TrEnh
VaxHiRisk
VaxMass
VaxYoung

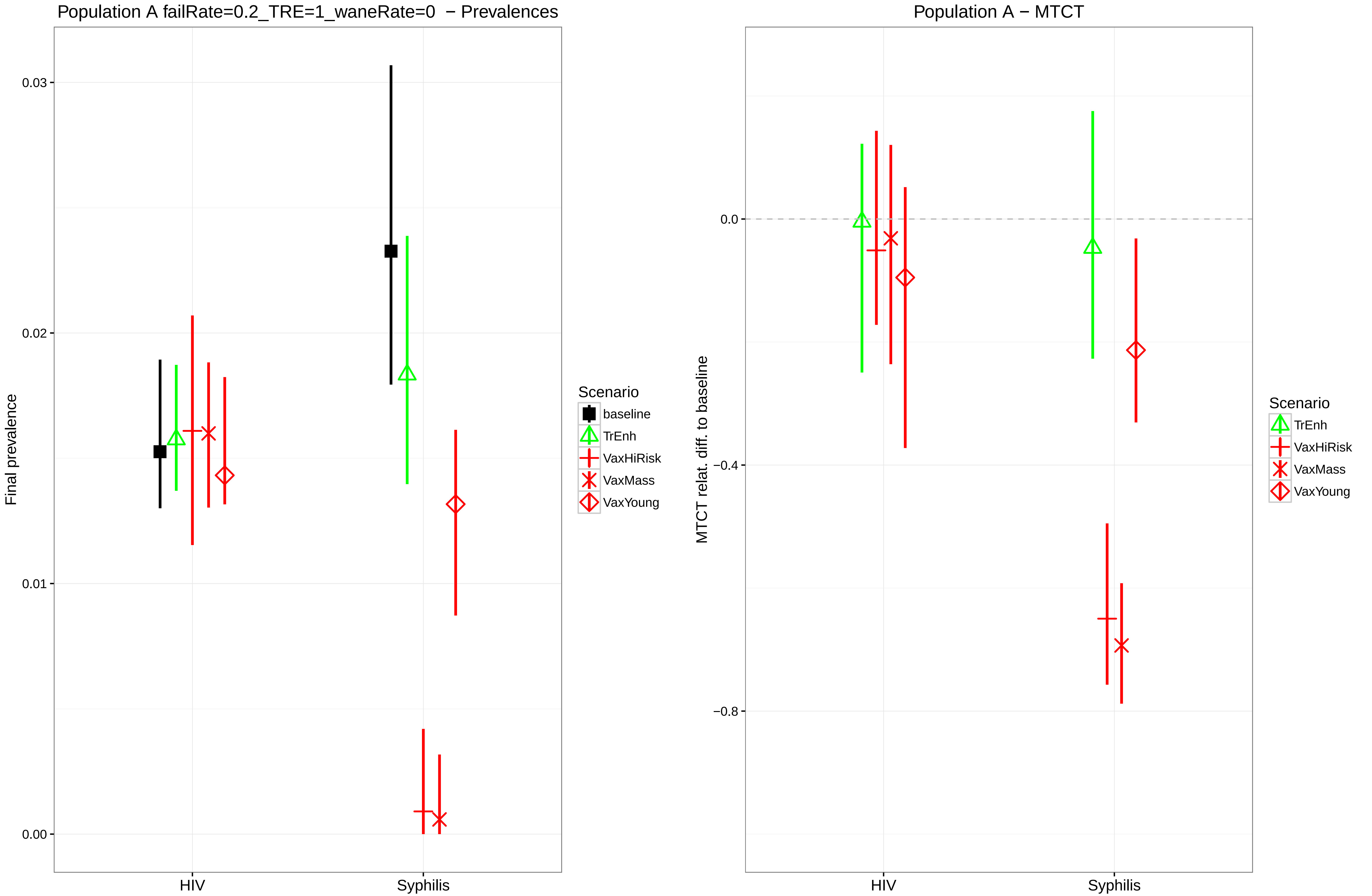
Population A failRate=0.2_TRE=1_waneRate=0 − Prevalences
Final prevalence
0.03
0.02
0.01
0.00
HIV
Syphilis
Scenario
baseline
TrEnh
VaxHiRisk
VaxMass
VaxYoung
Population A − MTCT
MTCT relat. diff. to baseline
0.0
−0.4
−0.8
HIV
Syphilis
Scenario
TrEnh
VaxHiRisk
VaxMass
VaxYoung

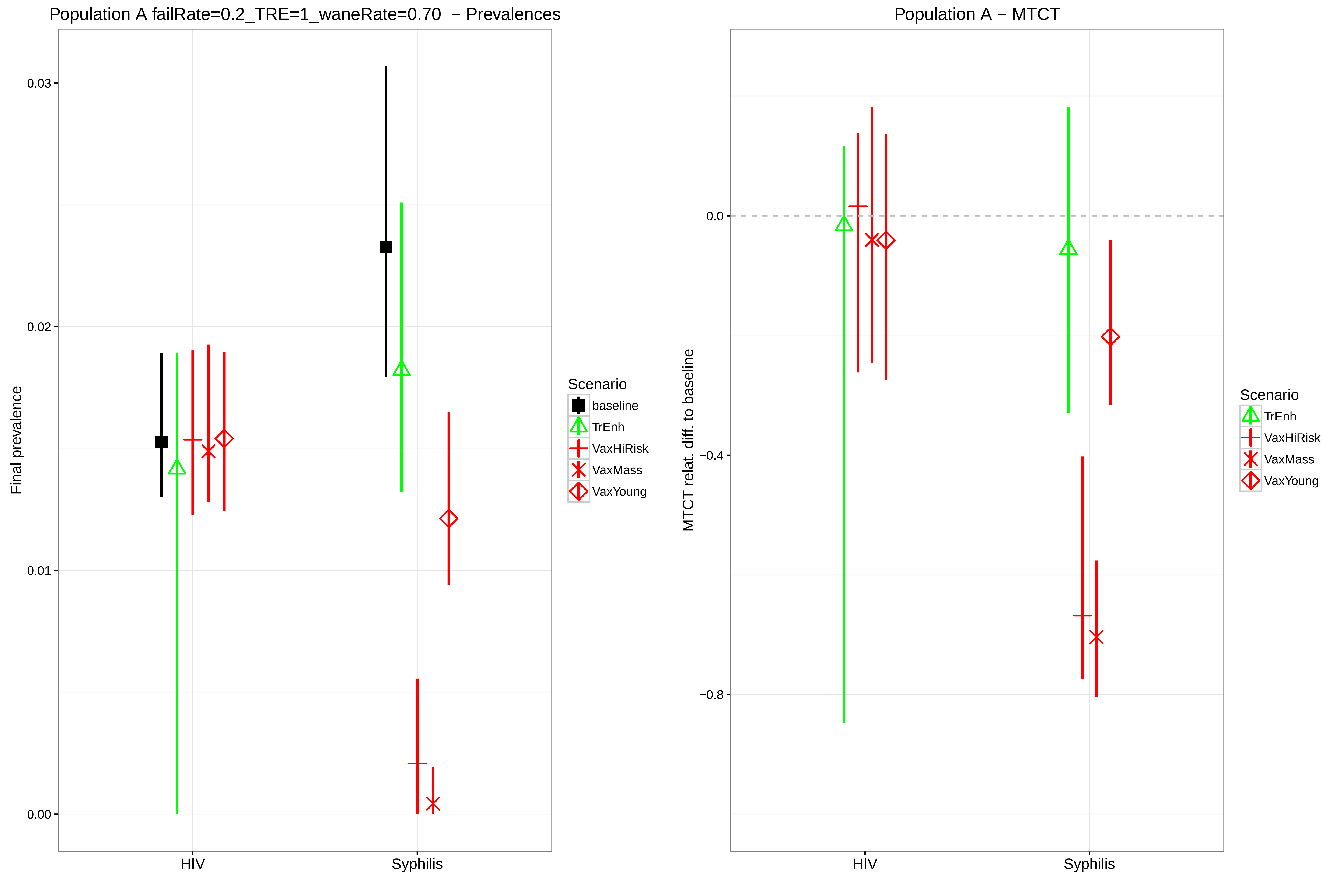
Population A failRate=0.2_TRE=1_waneRate=0.70 − Prevalences
Final prevalence
0.03
0.02
0.01
0.00
HIV
Syphilis
Scenario
baseline
TrEnh
VaxHiRisk
VaxMass
VaxYoung
Population A − MTCT
MTCT relat. diff. to baseline
0.0
−0.4
−0.8
HIV
Syphilis
Scenario
TrEnh
VaxHiRisk
VaxMass
VaxYoung

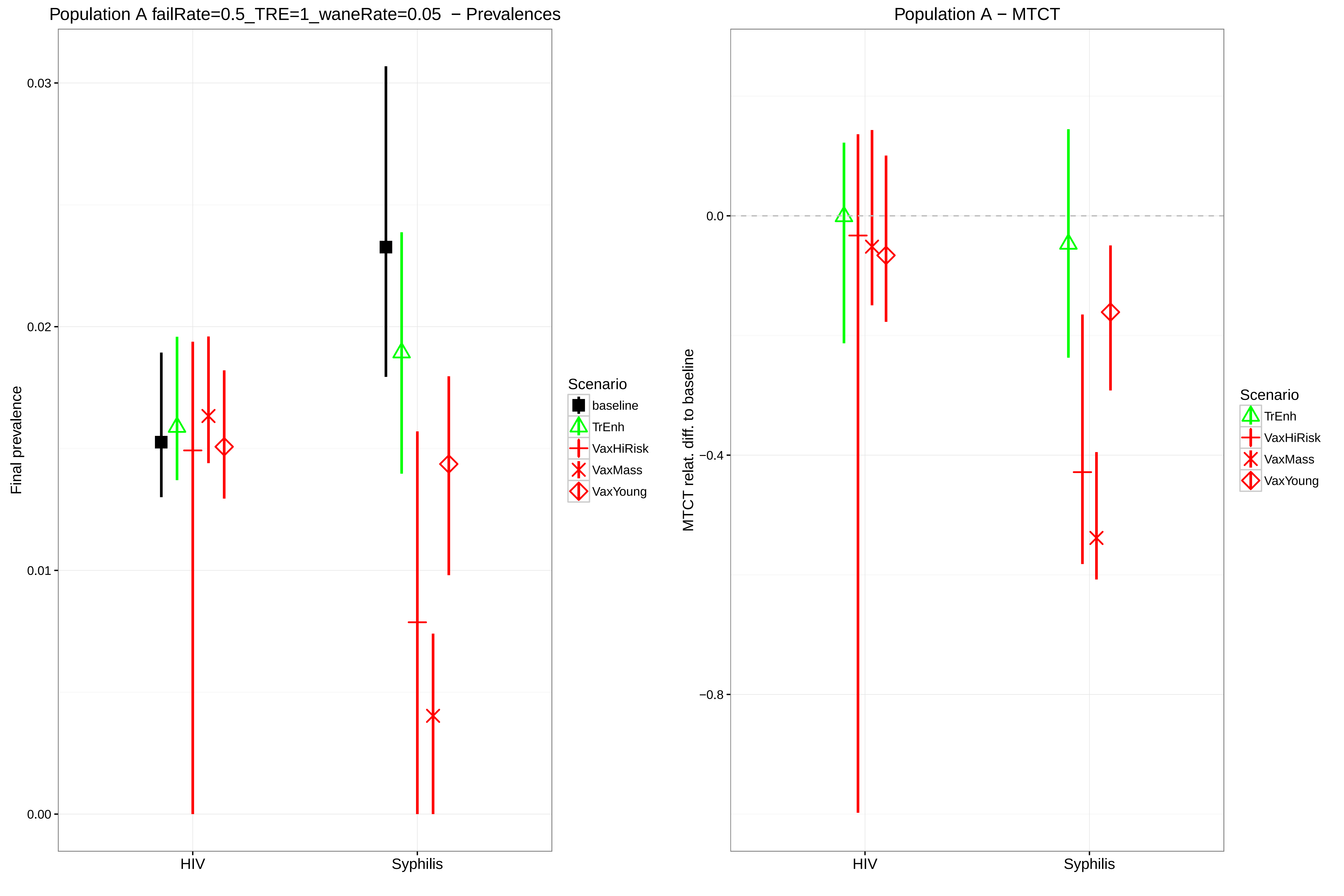
Population A failRate=0.5_TRE=1_waneRate=0.05 − Prevalences
Final prevalence
0.03
0.02
0.01
0.00
HIV
Syphilis
Scenario
baseline
TrEnh
VaxHiRisk
VaxMass
VaxYoung
Population A − MTCT
MTCT relat. diff. to baseline
0.0
−0.4
−0.8
HIV
Syphilis
Scenario
TrEnh
VaxHiRisk
VaxMass
VaxYoung

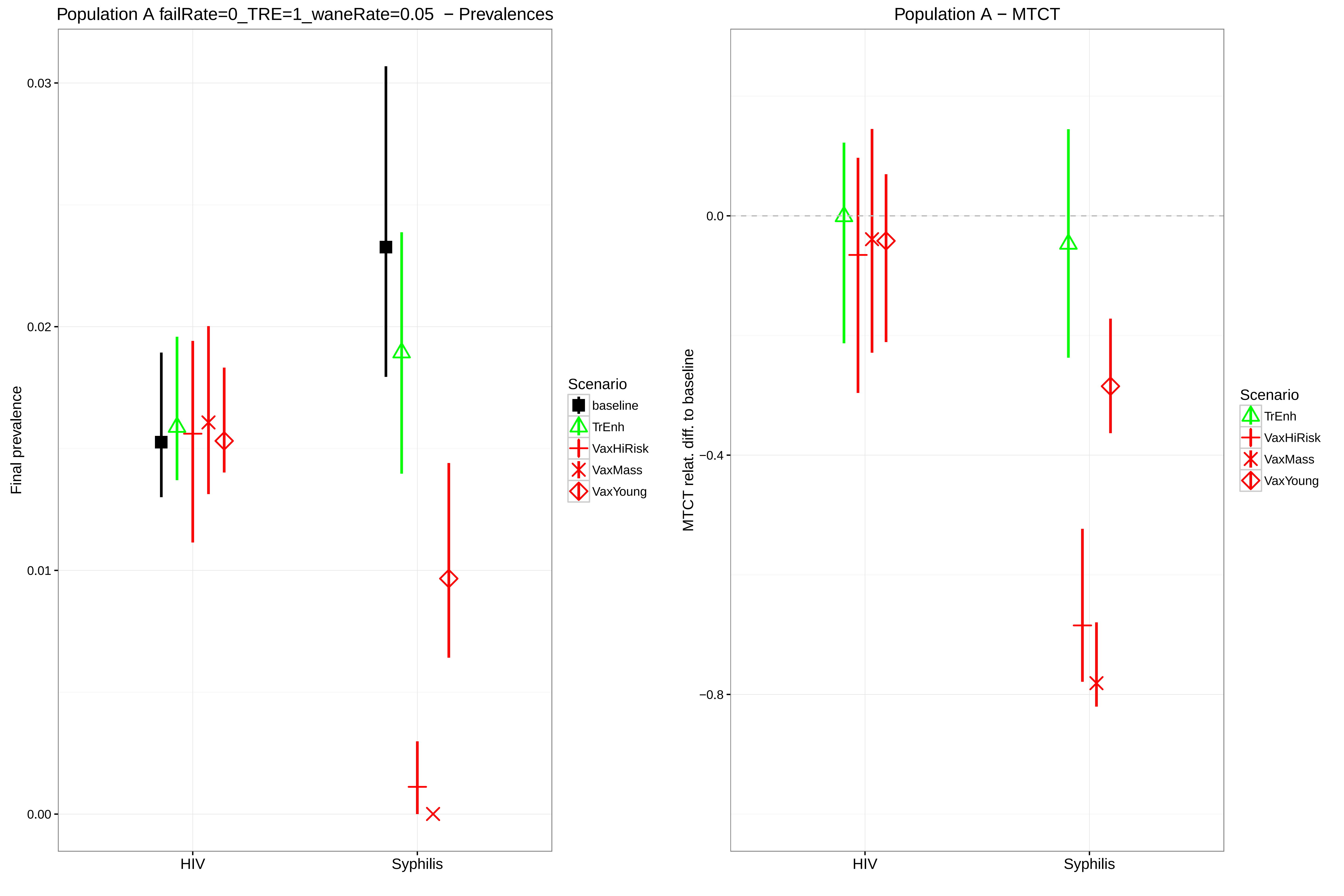
Population A failRate=0_TRE=1_waneRate=0.05 − Prevalences
Final prevalence
0.03
0.02
0.01
0.00
HIV
Syphilis
Scenario
baseline
TrEnh
VaxHiRisk
VaxMass
VaxYoung
Population A − MTCT
MTCT relat. diff. to baseline
0.0
−0.4
−0.8
HIV
Syphilis
Scenario
TrEnh
VaxHiRisk
VaxMass
VaxYoung

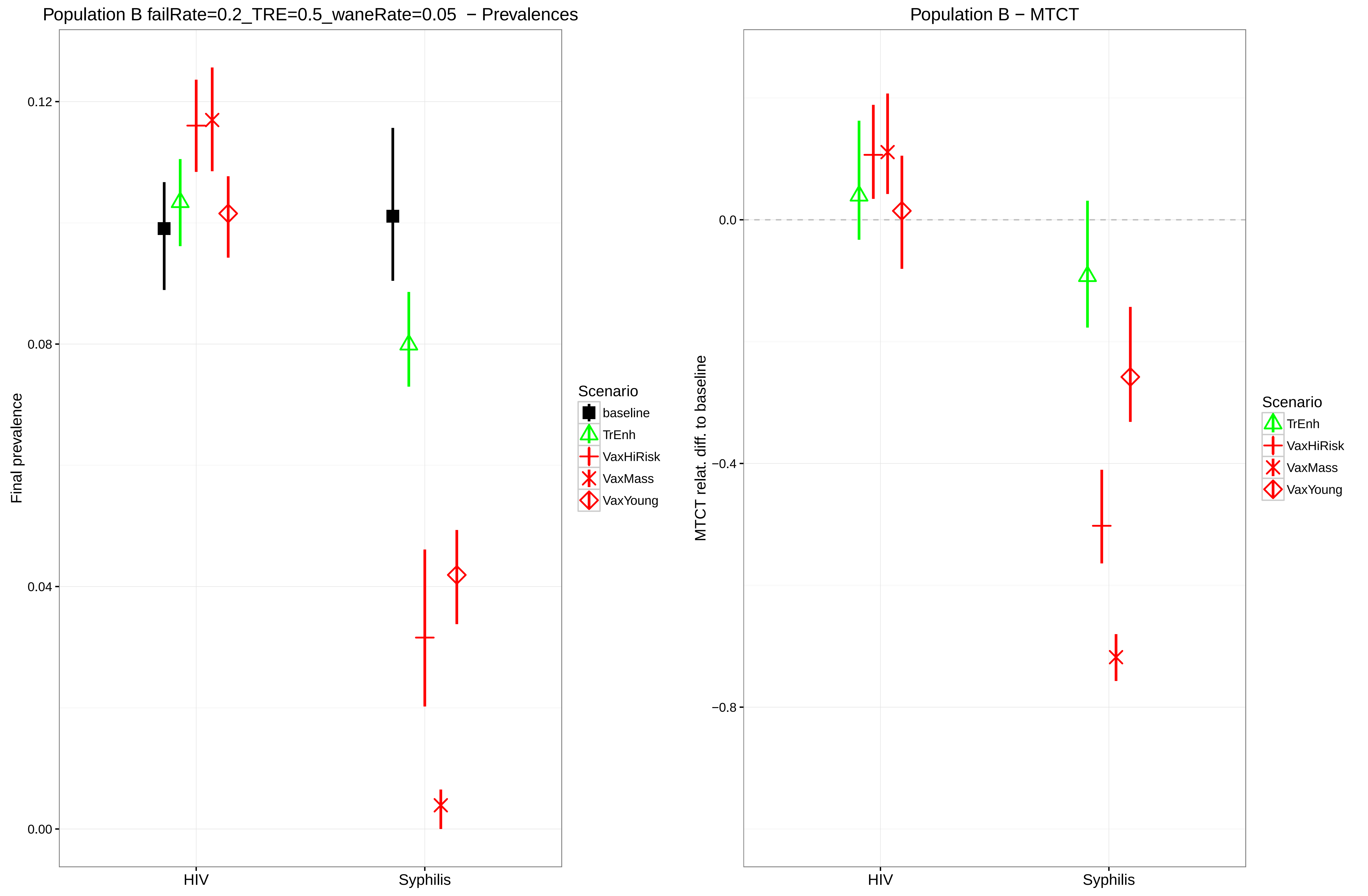
Population B failRate=0.2_TRE=0.5_waneRate=0.05 − Prevalences
Final prevalence
0.12
0.08
0.04
0.00
HIV
Syphilis
Scenario
baseline
TrEnh
VaxHiRisk
VaxMass
VaxYoung
Population B − MTCT
MTCT relat. diff. to baseline
0.0
−0.4
−0.8
HIV
Syphilis
Scenario
TrEnh
VaxHiRisk
VaxMass
VaxYoung

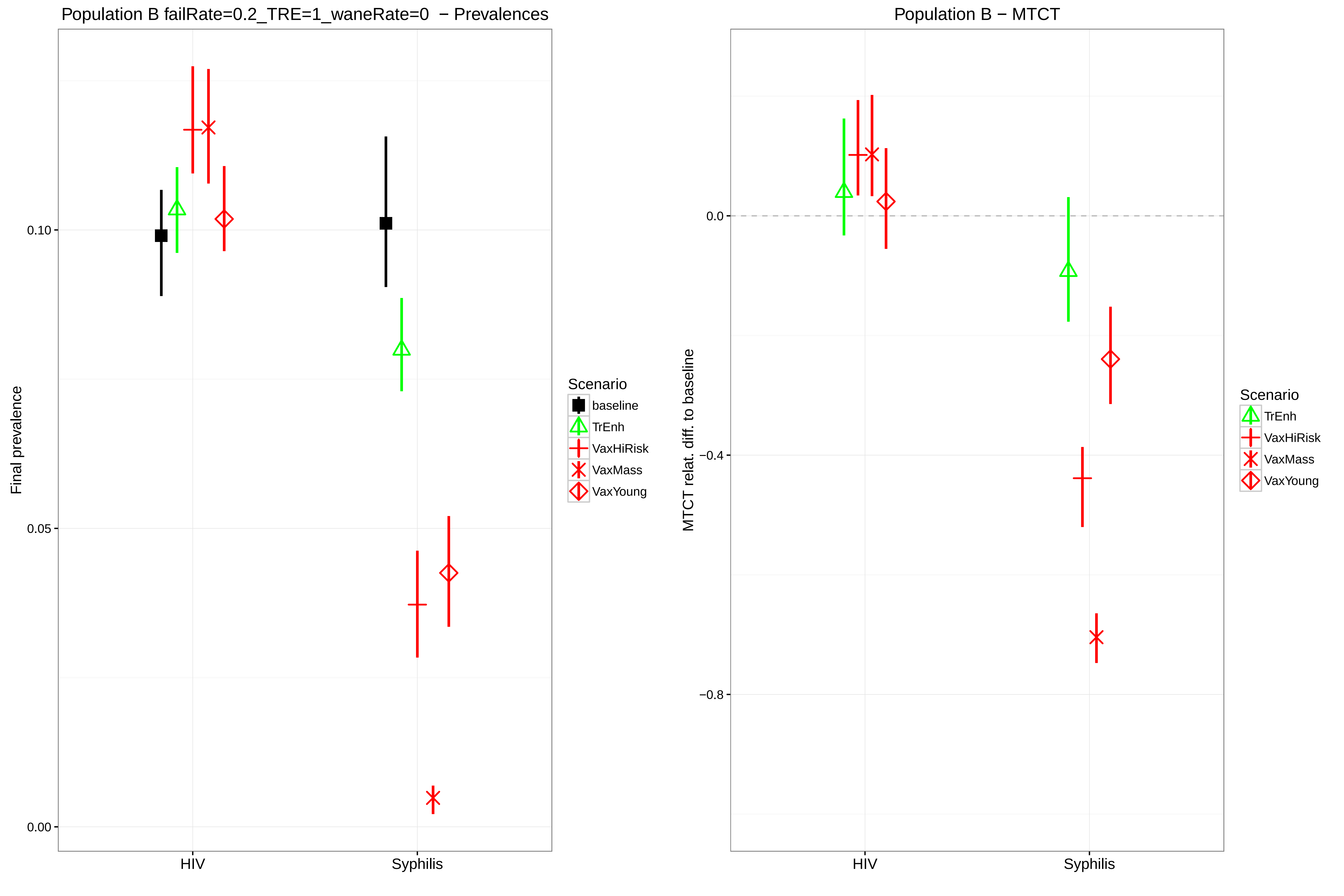

Population B failRate=0.2_TRE=1_waneRate=0 − Prevalences
Final prevalence
0.10
0.05
0.00
HIV
Syphilis
Scenario
baseline
TrEnh
VaxHiRisk
VaxMass
VaxYoung
Population B − MTCT
MTCT relat. diff. to baseline
0.0
−0.4
−0.8
HIV
Syphilis
Scenario
TrEnh
VaxHiRisk
VaxMass
VaxYoung

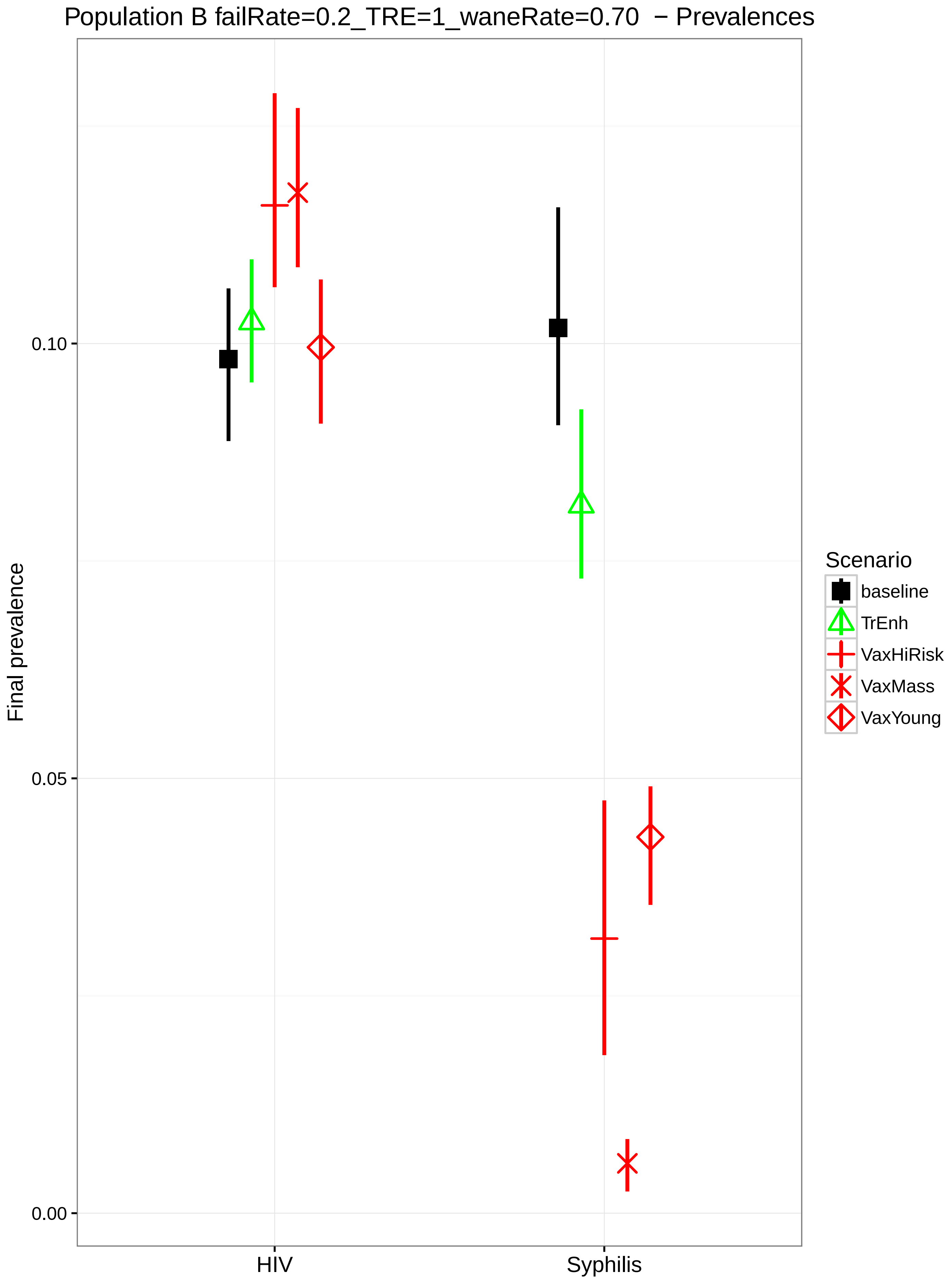

Population B failRate=0.2_TRE=1_waneRate=0.70 − Prevalences
Final prevalence
0.10
0.05
0.00
HIV
Syphilis
Scenario
baseline
TrEnh
VaxHiRisk
VaxMass
VaxYoung

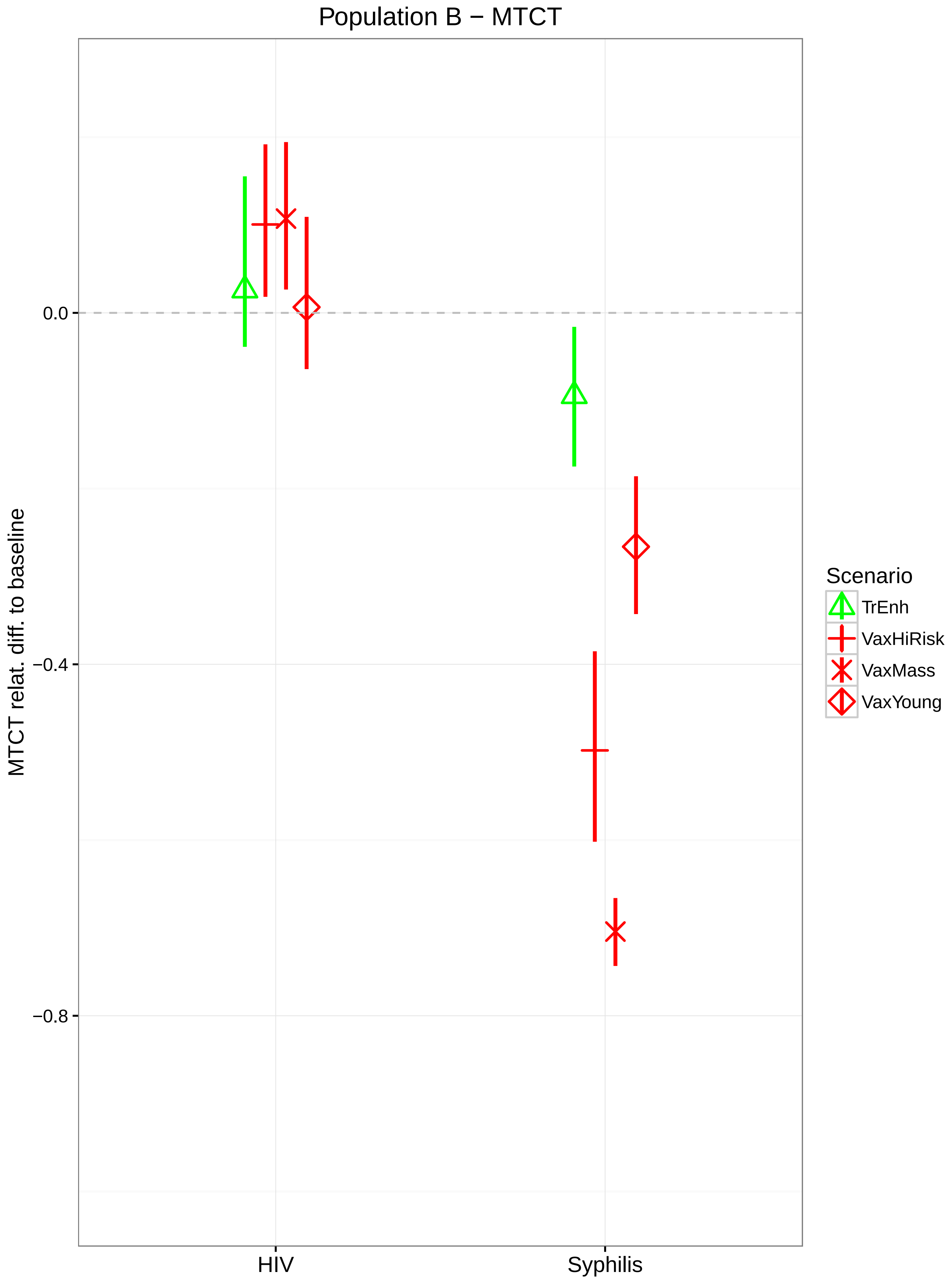

Population B − MTCT
MTCT relat. diff. to baseline
0.0
−0.4
−0.8
HIV
Syphilis
Scenario
TrEnh
VaxHiRisk
VaxMass
VaxYoung

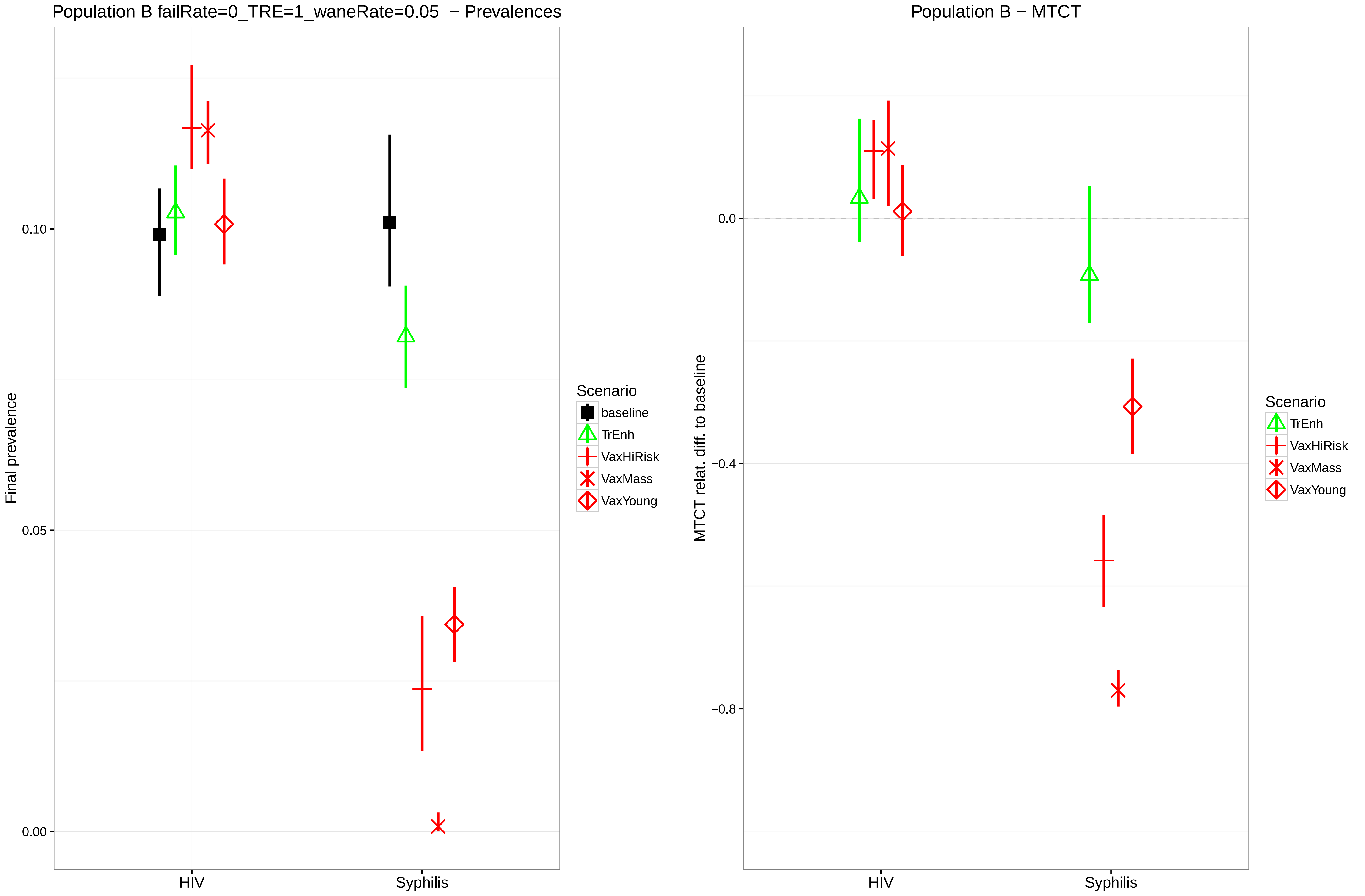
Population B failRate=0_TRE=1_waneRate=0.05 − Prevalences
Final prevalence
0.10
0.05
0.00
HIV
Syphilis
Scenario
baseline
TrEnh
VaxHiRisk
VaxMass
VaxYoung
Population B − MTCT
MTCT relat. diff. to baseline
0.0
−0.4
−0.8
HIV
Syphilis
Scenario
TrEnh
VaxHiRisk
VaxMass
VaxYoung

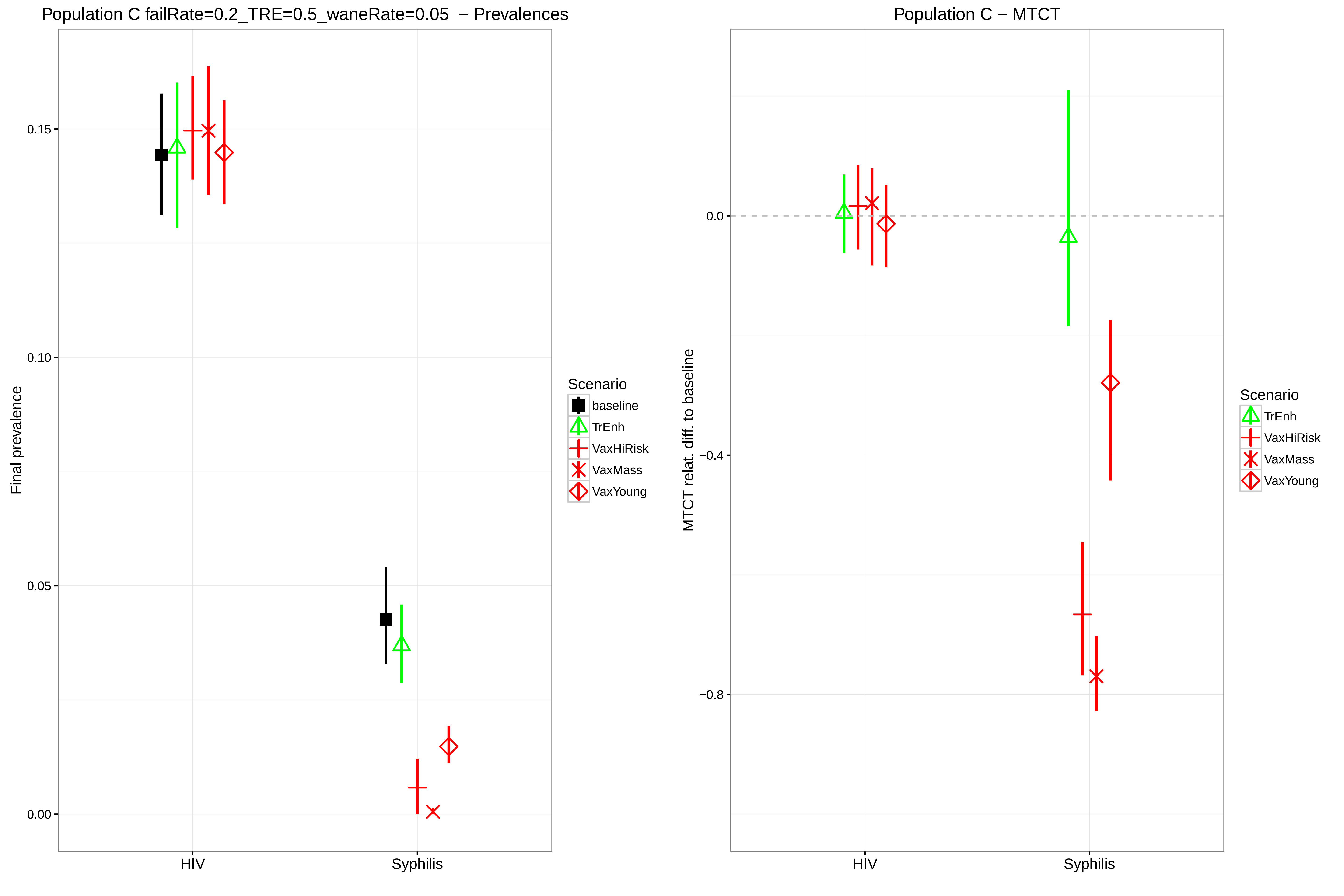
Population C failRate=0.2_TRE=0.5_waneRate=0.05 − Prevalences
Final prevalence
0.00
0.05
0.10
0.15
HIV
Syphilis
Scenario
baseline
TrEnh
VaxHiRisk
VaxMass
VaxYoung
Population C − MTCT
MTCT relat. diff. to baseline
0.0
−0.4
−0.8
HIV
Syphilis
Scenario
TrEnh
VaxHiRisk
VaxMass
VaxYoung

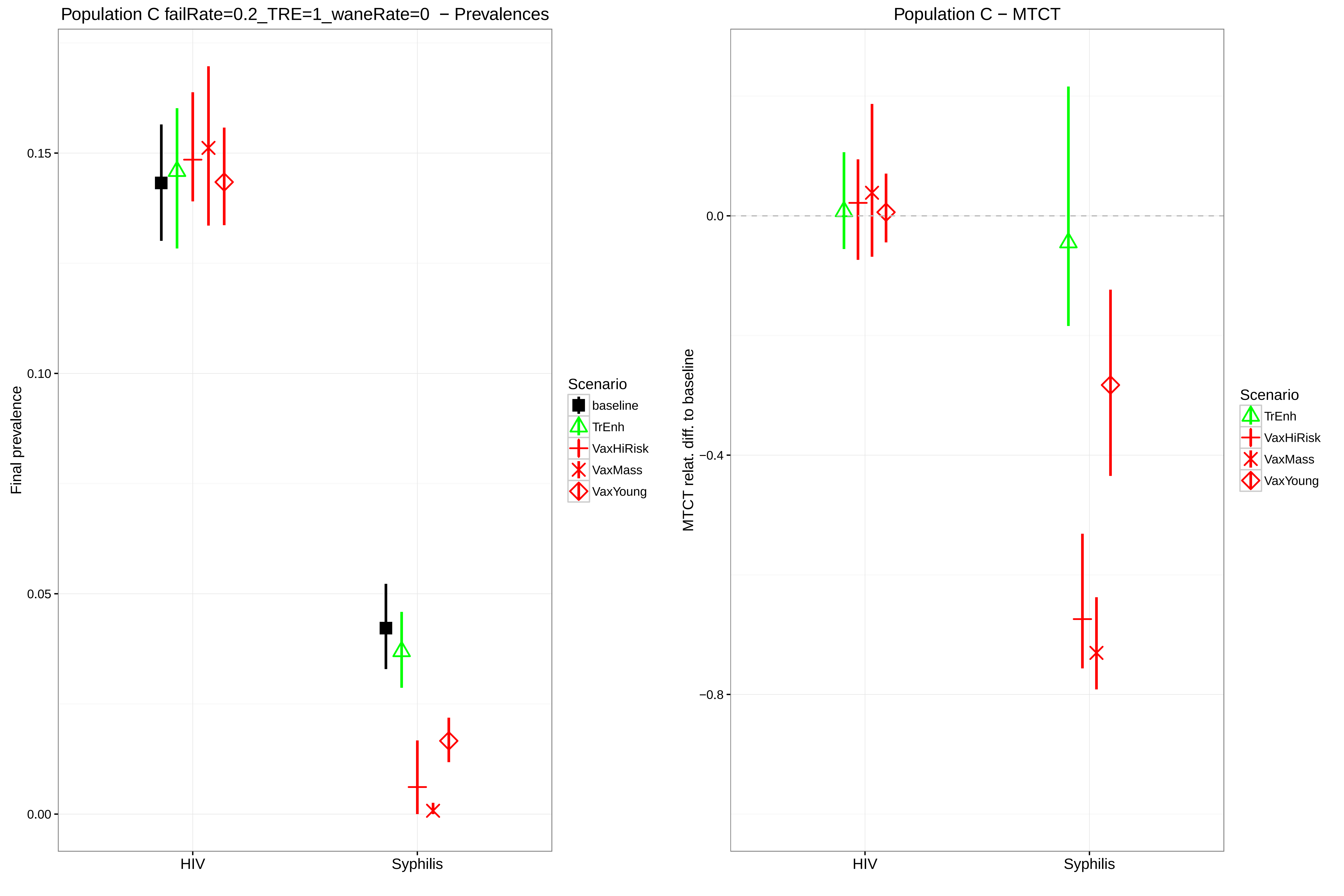

Population C failRate=0.2_TRE=1_waneRate=0 − Prevalences
Final prevalence
0.15
0.10
0.05
0.00
HIV
Syphilis
Scenario
baseline
TrEnh
VaxHiRisk
VaxMass
VaxYoung
Population C − MTCT
MTCT relat. diff. to baseline
0.0
−0.4
−0.8
HIV
Syphilis
Scenario
TrEnh
VaxHiRisk
VaxMass
VaxYoung

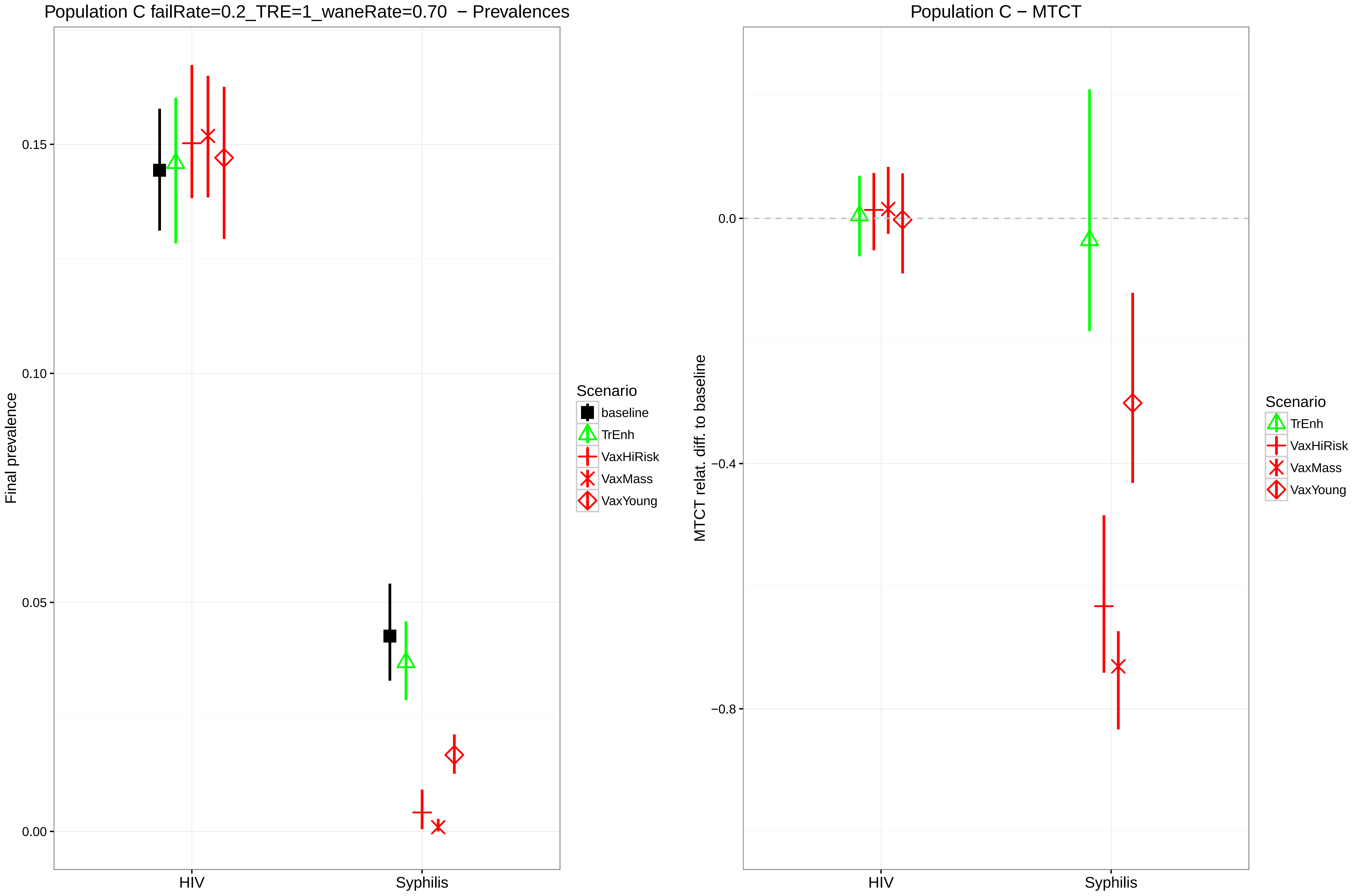

Population C failRate=0.2_TRE=1_waneRate=0.70 − Prevalences
Final prevalence
0.15
0.10
0.05
0.00
HIV
Syphilis
Scenario
baseline
TrEnh
VaxHiRisk
VaxMass
VaxYoung
Population C − MTCT
MTCT relat. diff. to baseline
0.0
−0.4
−0.8
HIV
Syphilis
Scenario
TrEnh
VaxHiRisk
VaxMass
VaxYoung

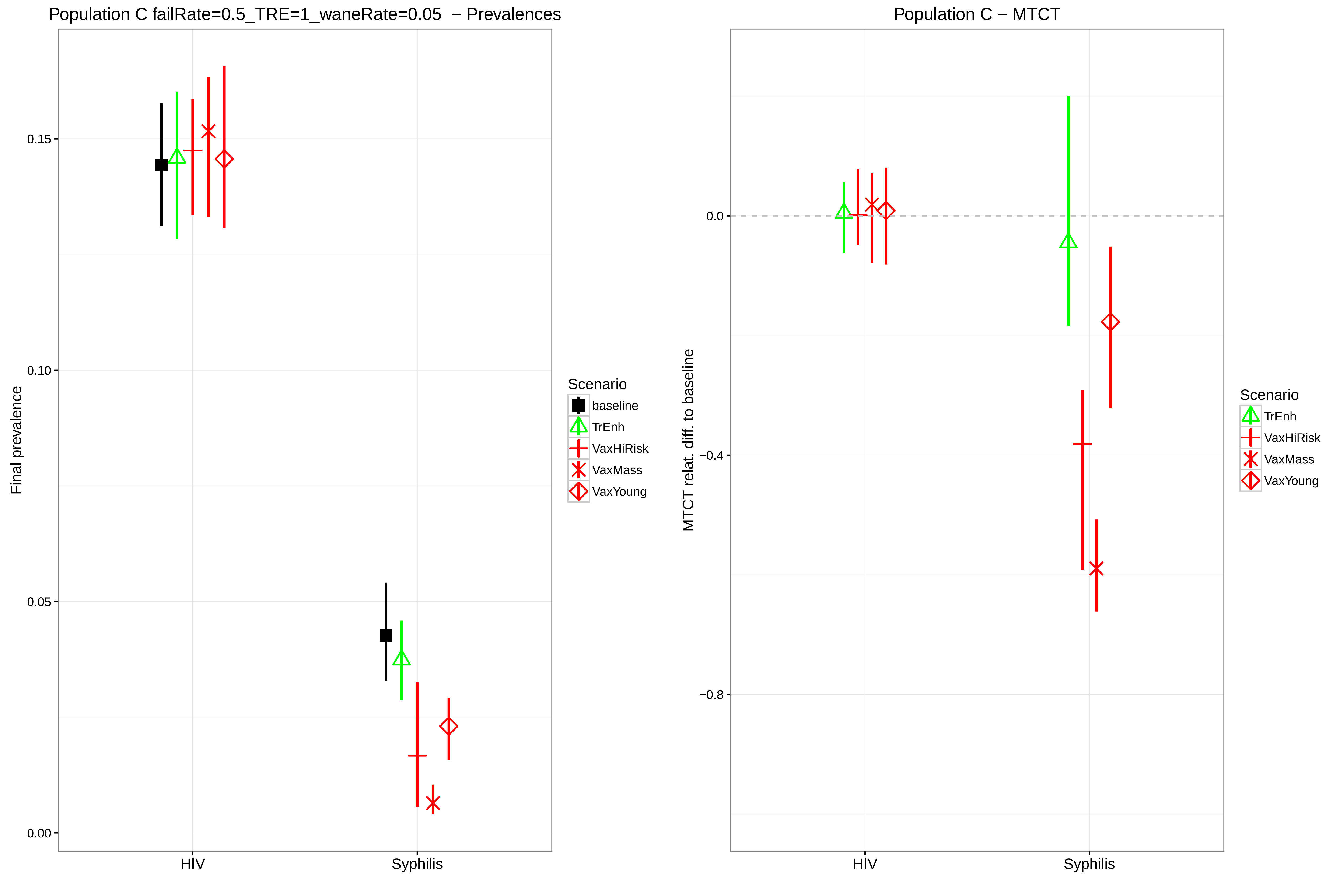

Population C failRate=0.5_TRE=1_waneRate=0.05 − Prevalences
Final prevalence
0.15
0.10
0.05
0.00
HIV
Syphilis
Scenario
baseline
TrEnh
VaxHiRisk
VaxMass
VaxYoung
Population C − MTCT
MTCT relat. diff. to baseline
0.0
−0.4
−0.8
HIV
Syphilis
Scenario
TrEnh
VaxHiRisk
VaxMass
VaxYoung

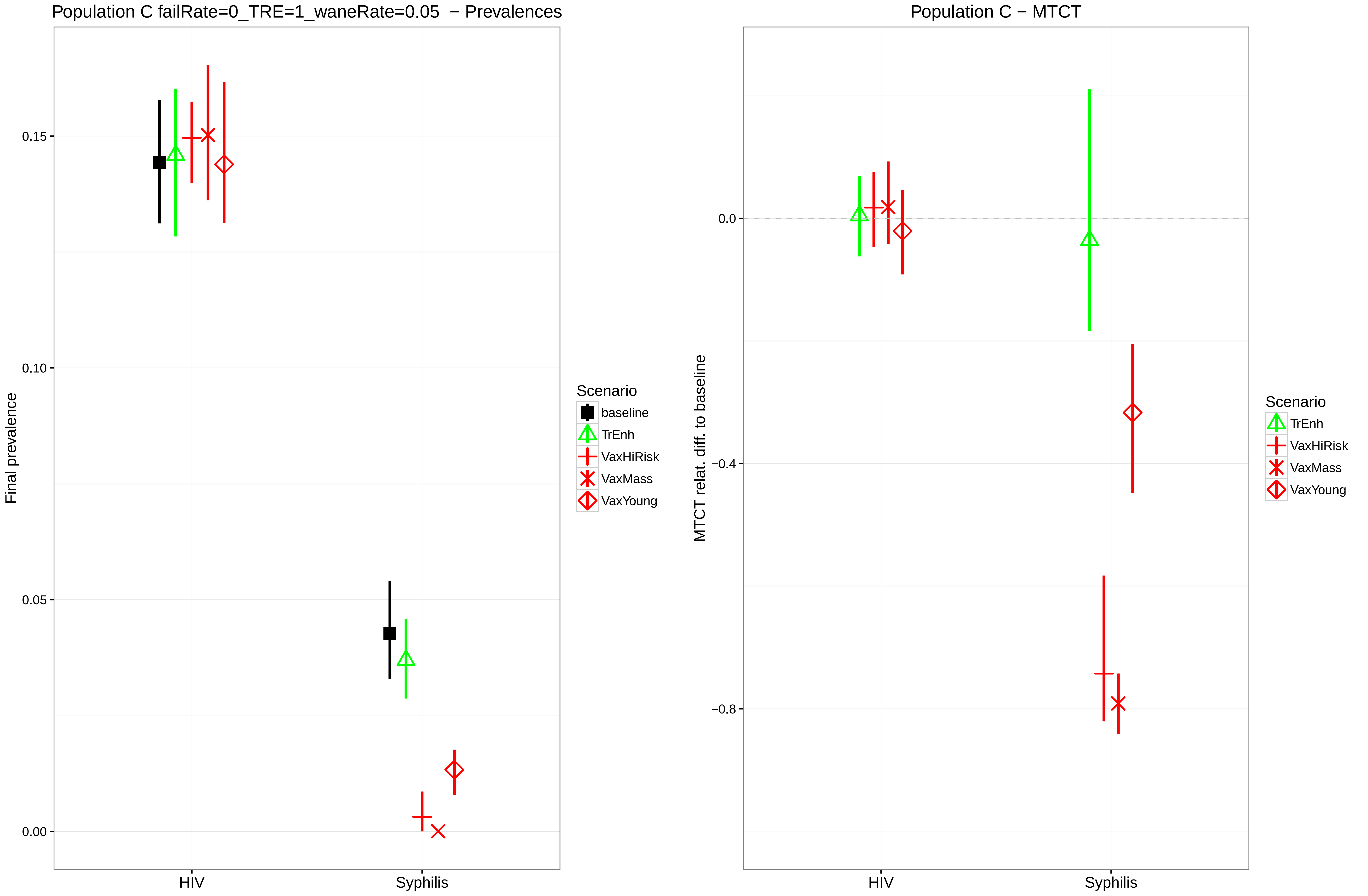

Population C failRate=0_TRE=1_waneRate=0.05 − Prevalences
Final prevalence
0.15
0.10
0.05
0.00
HIV
Syphilis
Scenario
baseline
TrEnh
VaxHiRisk
VaxMass
VaxYoung
Population C − MTCT
MTCT relat. diff. to baseline
0.0
−0.4
−0.8
HIV
Syphilis
Scenario
TrEnh
VaxHiRisk
VaxMass
VaxYoung
